# Quantitative proteome dynamics across embryogenesis in a model chordate

**DOI:** 10.1016/j.isci.2024.109355

**Published:** 2024-02-29

**Authors:** Alexander N. Frese, Andrea Mariossi, Michael S. Levine, Martin Wühr

**Affiliations:** 1 Lewis-Sigler Institute for Integrative Genomics, Princeton University, Princeton, NJ, USA; 2 Department of Molecular Biology, Princeton University, Princeton, NJ, USA

**Keywords:** Animals, Embryology, Evolutionary developmental biology, Proteomics, Transcriptomics

## Abstract

The evolution of gene expression programs underlying the development of vertebrates remains poorly characterized. Here, we present a comprehensive proteome atlas of the model chordate *Ciona*, covering eight developmental stages and ∼7,000 translated genes, accompanied by a multi-omics analysis of co-evolution with the vertebrate *Xenopus*. Quantitative proteome comparisons argue against the widely held hourglass model, based solely on transcriptomic profiles, whereby peak conservation is observed during mid-developmental stages. Our analysis reveals maximal divergence at these stages, particularly gastrulation and neurulation. Together, our work provides a valuable resource for evaluating conservation and divergence of multi-omics profiles underlying the diversification of vertebrates.

## Highlights

• Resource of absolute concentration for ∼6,000 proteins in the *Ciona* egg
• Comprehensive quantitative analysis of ∼7,000 proteins during *Ciona* development
• Embryonic protein dynamics are evolutionarily more conserved than those of mRNA
• Cross-species protein dynamic comparison supports an inverse hourglass model

## Graphical abstract

**Figure:**
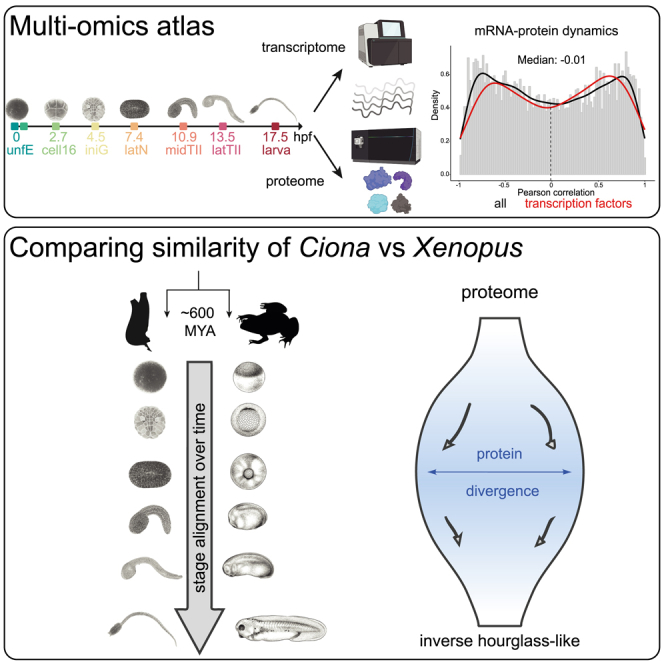

## Introduction

Embryonic development progresses through a series of cellular states, each defined by distinct changes in mRNA and protein levels. Optimal cellular functionality depends on precise control of gene expression and correct protein concentrations.^1^^,^^2^^,^^3^ However, (1) accurately measuring protein concentrations and (2) understanding the mechanisms governing cellular proteostasis remain a significant challenge.

While transcriptomic studies often rely on mRNA levels to predict protein concentrations, the key determinants of cellular functionality and phenotype, numerous studies have reported weak correlations between the two, challenging their reliability as proxies for each other.^4^^,^^5^^,^^6^^,^^7^ This disparity is influenced by the stochastic nature of mRNA transcription, translation, and degradation and becomes particularly pronounced for dynamic cellular transitions during embryogenesis.^8^^,^^9^^,^^10^^,^^11^ Thus, mRNA levels are not necessarily predictive of protein concentrations, which prompts a shift toward applying more comprehensive proteome-wide analyses.

Proteomic methods provide an accurate measurement of protein abundance but have been historically limited by technical challenges.^12^ Recent advancements in quantitative multiplexed mass spectrometry (MS) have significantly enhanced the sensitivity and precision of these measurements, expanding our capacity to map the cellular proteome in detail.^13^^,^^14^^,^^15^^,^^16^^,^^17^^,^^18^ Applying these techniques to the study of vertebrate embryos still presents considerable challenges. The system’s complexity, high cell numbers, and substantial yolk content, which affects detection of moderate and low abundance proteins, have limited the coverage and scope of these analyses.^4^^,^^19^^,^^20^^,^^21^^,^^22^^,^^23^ Urochordates are the nearest extant relatives to vertebrates and share several morphological and genomic traits.^24^ In particular, *Ciona* has numerous experimental advantages like small size, low cell number, stereotyped cell lineages, rapid and comparatively simple development with experimental tractable embryogenesis, and a compact genome that is not complicated by the gene duplication events accompanying the advent of the vertebrates.^25^ Additionally, *Ciona* retains conservation of non-coding elements, macrosynteny, and microsynteny with chordates, making it an ideal model for studying the evolution of vertebrate developmental processes.^26^^,^^27^^,^^28^^,^^29^^,^^30^^,^^31^

While *Ciona* lacks the complex specializations and innovations characteristic of vertebrates, it has nonetheless advanced our understanding of the morphogenesis of basic chordate tissues such as the muscles, heart and notochord, as well as the evolution of key vertebrate processes such as neural crest.^32^^,^^33^^,^^34^^,^^35^^,^^36^^,^^37^^,^^38^^,^^39^^,^^40^ The assembly of the *Ciona* genome^25^ represented a significant landmark that enabled a variety of transcriptomics, epigenomics, and single-cell studies.^41^^,^^42^^,^^43^^,^^44^^,^^45^^,^^46^^,^^47^ Here, we extend these large-scale datasets through the use of quantitative proteomics methods.

The evolution of gene expression and its role in morphological innovations have been studied primarily by comparative transcriptomics.^48^^,^^49^^,^^50^^,^^51^ These studies point toward a 'phylotypic period’ in vertebrates, whereby gene expression is most similar across different species during mid-embryogenesis or pharyngula stage, the “hourglass” model.^52^ However, comparisons with non-vertebrate chordates such as tunicates and cephalochordates are not entirely consistent with the hourglass.^53^ This suggests potential divergent developmental pathways or an earlier onset of conservation as compared with vertebrates. For example, in amphioxus this conservation aligns with the earlier neurula stage.^54^ In fact, extending comparative analysis to invertebrates, suggests an inverse hourglass model with increased conservation during early and late developmental stages rather than in the middle of development.^55^^,^^56^^,^^57^ This model implies a bottleneck in developmental pathways, potentially influencing the emergence of species-specific traits. The effectiveness of these comparative analyses require careful consideration of phylogenetic distances, species diversity, embryonic stages, and gene sets compared.^58^ Several studies stress limitations of simplistic pairwise comparisons, robust testing of null hypotheses, and the challenge in balancing phylogenetic distances, which can be too short among closely related species or too extensive when the comparisons are made between vertebrates and invertebrates or across multiple phyla.^51^^,^^53^^,^^58^

A major limitation of the earlier studies is the reliance of transcriptome datasets to infer the dynamics of gene activities.^59^ Recent reports suggest significant disparities in mRNA and protein levels.^4^^,^^5^^,^^6^^,^^60^ In this study we re-examine similarity of embryos at various developmental stages with comparisons of both transcriptome and proteome datasets. Proteomic studies offer a novel perspective in cross-species comparisons by quantifying protein conservation patterns, which are the primary executors of most cellular functions.^61^

Here, we use state-of-the-art proteomics to quantify proteins in unfertilized *Ciona* eggs and to track proteomic changes throughout embryogenesis, revealing that the embryonic proteome accounts for at least half of the genome’s protein-coding capacity. We create a detailed genome-wide dataset that shows precise measurement of protein kinetics and their association to key developmental processes such as fertilization, maternal-to-zygotic transition (MZT), gastrulation, and the formation of larval tissues. Further, we integrated these data with corresponding transcriptome information and carried out inter-species comparisons between *Ciona* and *Xenopus laevis*, the African clawed frog. We discuss the implications of these studies with respect to the conservation and divergence of genetic activities during chordate evolution and reconsider the hourglass model of development.

## Results and discussion

### Adapting proteomics for the analysis of *Ciona* eggs and embryos

Mass spectrometry-based proteomics (MS) is a versatile tool for studying a variety of biological processes, although new model systems often require method adaptations. Key areas needing optimization include sample preparation and the reference proteome. Analyzing eggs and early embryos is often challenging due to the high yolk content. For instance, in *Xenopus*, yolk constitutes ∼90% of egg protein content, limiting the depth of proteomics analyzes.^62^ Researchers usually remove yolk through centrifugation after lysis of eggs or embryos.^63^^,^^64^^,^^65^ However, when we analyzed *Ciona* egg lysates via Coomassie-stained gels, we found no exceptionally dominant protein band (Figure S1A), allowing us to analyze *Ciona* samples by MS without yolk removal. Another concern in proteomics is the quality of the protein reference database. For widely used models such as humans, mice, or yeast, this is typically derived from the genome. However, the quality of the genome for non-canonical model organisms is often poor, thereby severely limiting the proteins that can be identified via MS. A better reference database can be generated based on mRNA-seq data.^64^^,^^66^ Accordingly, we first evaluated the quality of the latest *Ciona* genome by benchmarking it against a genome-free protein reference database, which we generated from available RNA-seq datasets (Figure S1B).^39^^,^^67^^,^^68^^,^^69^ Upon comparison, the RNA-seq based reference database clearly outperformed Uniprot^70^ and the previous genome annotations (KH-2013 and KY19),^71^ but increased peptide coverage by only 5% compared to the most recent KY21 annotation (Figures S1C and S1D).^72^ We decided to accept the modest decrease in identified peptides for the ease of annotation offered by the genome assembly and proceeded to use the KY21 genome as our primary reference for the remainder of this study.

Further examination of peptides identified using our genome-free database revealed mis-annotated gene coding sequences, mis-positioned intercistronic regions, and discrepancies in selenoprotein sequences present in the KY21 proteome (Figure S1E).^73^^,^^74^^,^^75^ We believe that our analysis is a step forward in improving the accuracy and completeness of the *Ciona* genome annotation and the potential of the proteome atlas to refine *Ciona* gene models and protein coding sequences. Collectively, our data reveals that the latest assembled *Ciona* genome, combined with the characteristics of its eggs and embryos, is highly suitable for proteomics studies, and supports *Ciona*’s potential as a valuable model system for proteomics investigation.

### Absolute protein abundance measurements in the unfertilized egg

**Figure 1:**
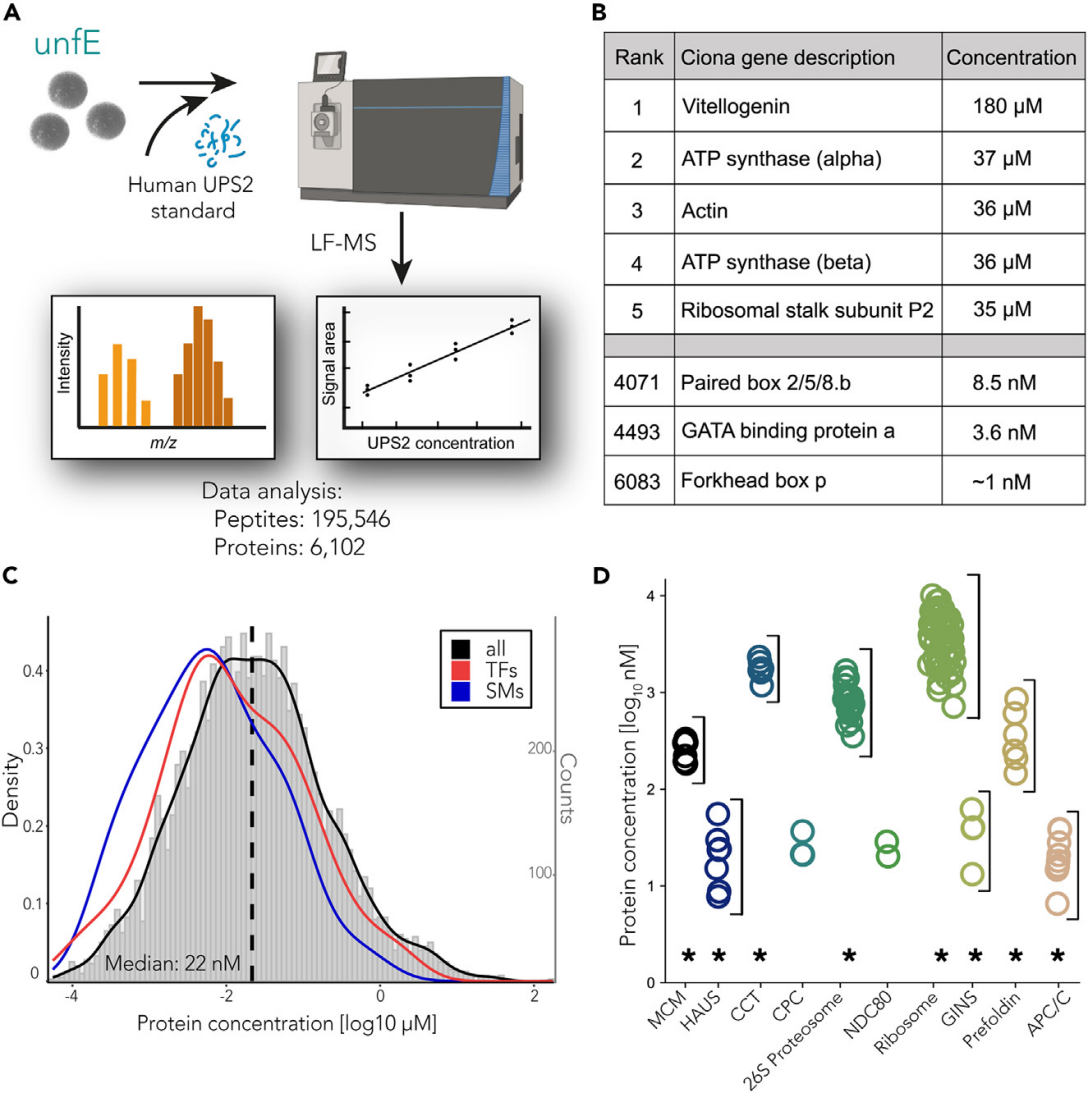
Absolute proteomics of the *Ciona* egg (A) Schematic of label-free proteomics utilized to determine absolute protein concentrations. Unfertilized *Ciona* eggs were lysed, and human proteins of known concentrations (UPS2) were added to the lysate as a reference standard. Following normalization as outlined in the materials and methods, we detect ∼195,000 peptides and estimate protein concentrations for ∼6,000 proteins. (B) Table of selected proteins in the unfertilized egg including the top 5 most abundant and some transcription factors important to embryonic development. (C) Histogram of all quantified proteins in the *Ciona* egg (gray) with superimposed kernel density estimates (KDE) of transcription factors (TFs - red) and signaling molecules (SMs - blue). Both TFs and SMs follow a distribution similar to the global egg proteome (black) but with a lower median concentration. The complete data is provided in Table S1. (D) Stoichiometries of protein complexes. Concentrations of subunits from a shared protein complex display comparable values and show typically a statistically different distribution than the entire proteome (∗p < 0.01, two-way ANOVA with Tukey’s multiple-comparisons test).

The mature egg contains an array of maternal proteins required for fertilization, transition to zygotic transcription, and the early stages of embryogenesis.^76^^,^^77^^,^^78^^,^^79^ Given that many of these proteins remain unidentified, incorporating a proteomic approach was the logical next step. We estimate the absolute concentrations of proteins in the unfertilized egg using MS1 precursor intensity in a deep label-free analysis.^64^ Altogether, we quantified the abundance of 6,102 proteins, after collapsing isoforms (Figure 1A; Table S1), thereby expanding the number of known proteins by an additional 5,058 entries compared to the previous proteomic investigation of the *Ciona* egg.^80^ Nearly 90% of identified proteins are supported by at least two peptides, and the mean sequence coverage is 21% (Figure S1F).

As expected, the most abundant protein is Vitellogenin (yolk protein), followed by ATP synthase subunits, actin, and a 60S ribosomal subunit (Figure 1B).^81^ The analysis spans approximately eight orders of magnitude, covering 95 transcription factors (TFs) and 46 signaling molecules (SMs) (Figure 1C). The median protein concentration is 22 nM. In contrast, the median concentrations of TFs and SMs are lower, 5.4 nM and 3.5 nM, respectively. Most of them are distributed toward the lower end of the concentration curve, aligning with reports from other systems, where it has been noted that these molecules can exert significant biological effects even at low concentrations, particularly in driving dynamic cellular processes such as differentiation.^82^ Among the identified TFs in the egg are known maternal factors such as Gata.a, Prd-B/Prdtun2, and Zeb (also known as Zinc Finger (C2H2)-33 or Ci-ZF266).^83^ Among the SMs, known maternal factors include β-Catenin, Eph.a/Eph1, Eph.b/Eph2, Raf/Raf1, Tll/Tolloid, Notch, and Numb.^83^^,^^84^^,^^85^ The interaction of these known maternal deposits have been reported to be essential to establish the first distinct spatial domains of gene expression that launch the gene regulatory networks controlling embryogenesis.^86^ Alongside these molecules, the proteomic landscape is characterized by an abundance of kinases and phosphatases, common regulatory components controlling the cell cycle and proliferation. Proteins indicative of posterior end markers (PEM), which include germline determinants and positional cues for the axial development of the embryo, are conspicuous components of the maternal proteome.^87^^,^^88^ These findings suggest a preparatory state for fertilization and subsequent developmental cascades. Furthermore, in addition to Vitellogenin, we observe a notable enrichment of metabolic components, emphasizing the importance of energy and nutritional reserve components supplied by the egg for the early stages of development. These proteins ensure that *Ciona* embryos, which do not feed before metamorphosis, have the necessary resources for successful settlement.

We next asked whether different subunits within the same protein complex are found at expected stoichiometric ratios. To this end, we mapped the proteins identified in the egg to known stable complexes from the CORUM database (Figure 1D).^89^ We observed overall comparatively tight distributions of subunits in most macromolecular complexes, such as MCM (involved in genomic DNA replication),^90^ CCT (playing a significant role in protein folding in the eukaryotic cytosol),^91^ the HAUS complex (essential for mitotic spindle assembly),^92^ and Prefoldin (chaperone proteins regulating correct protein folding).^93^ For all the complexes for which we detect more than two subunits, the distribution is significantly different from the distribution of the entire dataset (p < 0.01, two-way ANOVA with Tukey’s multiple-comparisons test) (Figure 1D).

Altogether, the proteomics of the unfertilized egg highlights intricate networks that anticipate subsequent developmental processes such as fertilization, spatial patterning, and hatching. The consistency of values obtained for different subunits of stoichiometric protein complexes corroborates the reliability of our data, providing a robust platform for future studies.

### A high-quality multi-omics atlas of *Ciona* development

**Figure 2:**
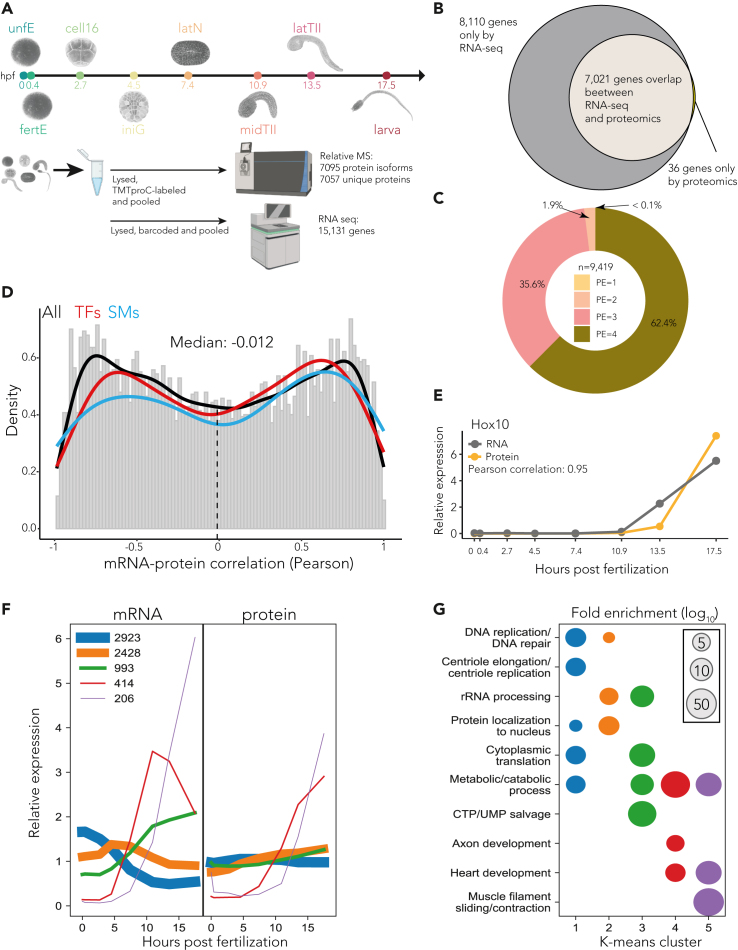
Proteome and RNA analyses during *Ciona* embryogenesis (A) Overview of the transcriptome and proteome time-course experiments. Staged embryos were collected at eight developmental stages, beginning with unfertilized egg (unfE), fertilized egg (fertE), 16-cell stage (cell-16), initial gastrula (iniG), late neurula (latN), middle tailbud II (midTII), late tailbud II (latTII), and hatching tadpole (larva). Each stage is represented by a unique color code, and abbreviation; both are kept consistent throughout the figures. Time indicates hours postfertilization (hpf). (B) Number and overlap of identified protein-coding genes in the transcriptome and proteome datasets. (C) Donut plot with the percentage of protein evidence categories from UniProt that are identified at the proteome level (9,419 entries). Evidence level: (1) protein evidence; (2) transcript evidence; (3) homology; (4) predicted. (D) Histogram of Pearson correlations between RNA and corresponding protein dynamics throughout *Ciona* development (gray). The lines represent kernel density estimates (KDE) for all genes (black), transcription factors (red), and signaling molecules (blue). Notably, mRNA dynamics correlate poorly with protein dynamics. n = 7021 pairs. (E) Example of high Pearson correlation between RNA and protein dynamics for the transcription factor Hox10. (F) K-means clustering used to classify RNA (left) and protein (right) dynamics for each gene during *Ciona* development. The thickness of the lines scales with the number represented in each cluster, as indicated in the legend. (G) GO term analysis used to discern the functional relevance of each of the clusters (indicated by matching colors) identified in F.

We next measured the dynamics of protein and mRNA abundances as the egg develops into a swimming tadpole. For this relative comparison analysis we combined accurate multiplexed proteome analysis (TMTproC)^14^ with RNA-seq on matching samples at eight key developmental stages. These stages span early embryonic development and include the maternal/zygotic transition, gastrulation, neurulation, tail elongation, and hatching of swimming tadpoles (Figure 2A), thereby encompassing all of the important developmental processes. Moreover, the parallel sampling of both modalities facilitates a direct comparison between RNA and protein expression.

Using this framework, we detected 7,095 protein isoforms encoded by 7,057 genes (Figure 2A; Table S2), representing 38% of the protein-coding genes annotated in the latest *Ciona* genome assembly.^72^ This accounts for approximately 50% of the expressed genes captured in RNA-seq analyses (Figure 2B; Table S3). This protein number is more than 10-fold greater than that reported in an earlier study, which identified 695 proteins across three sampled stages using two-dimensional gel electrophoresis and MALDI-TOF/MS.^81^ Our proteome marks a significant advancement in the quality of the UniProt database, which reports experimental evidence at the protein level (PE1) for less than 1% (21 out of 17,311 records). We cover 55% of the redundant UniProt entries, of which four had prior evidence at the PE1 level. Importantly, we confirmed protein products for an additional 9,415 entries previously undocumented at the protein level, categorized under evidence levels PE2–4 (Figure 2C). The new proteome dataset significantly expands the known proteomic landscape of *Ciona*.

### Descriptive analysis of proteomic data and RNA-seq atlas

For MS data, we applied a 1% false discovery rate with a target-decoy strategy^94^^,^^95^ (Figure S2A). We quantify a total of 62,471 peptides, the proteins with most identified peptides are Vitellogenin and Titin (Figure S2B). The median number of peptides per quantified protein is 5, with 84% of the proteome showing more than two peptides per protein (Figure S2B). The identified peptides correspond to 7,095 protein isoforms matching 7,057 unique proteins (Figure 1A). In 35 instances, the dataset enabled differentiation between 2 and 4 splice variants (Figure S2C). The poly(A) pulldown RNA-seq datasets cover an average of 10,727 ± 1,007 genes (mean ± s.d.), with high reproducibility of the biological replicates (Figures S3A–S3C). The number of detected genes steadily increases as development proceeds, reflecting an expanding gene expression repertoire (Figure S3D). However, post-zygotic genome activation (ZGA) at the 16-cell stage did not result in an increase in gene counts, likely due to the degradation of maternal mRNAs as previously observed in zebrafish development.^96^ The distribution of expression levels (transcripts per million, TPM) initially exhibited a bimodal pattern with peaks at very low and higher levels. As embryonic development proceeded, this distribution evolved into a more normal distribution (Figure S3E). These observations are consistent with the transition of bimodal distributions seen for homogeneous cell populations to a unimodal distribution for heterogeneous cell populations.^97^

### Temporal dynamics and tissue-specific patterns in the proteome atlas

In order to extend our analysis and systematically identify proteins that may influence differentiation programs, we categorized the proteins into eight distinct clusters based on their activity at various stages (Figure S4A) and performed gene ontology (GO) enrichment analysis on each gene cluster (Table S2). Cluster 1 genes exhibited the most stable dynamics, with proteins involved in translation, RNA processing, cell division, DNA organization, ribonucleoprotein complex formation, ribosome biogenesis, and transfer RNA (tRNA) activity. These are indicative of housekeeping functions. Cluster 2 genes, most abundant in unfertilized eggs, rapidly degrade following fertilization and are enriched for mRNA processing, single fertilization proteins, and small GTPase-mediated signal transduction, aligning with spindle assembly roles post-fertilization. They also have an abundance of maternal ribosomes preparing embryos for future development. Proteins in Cluster 3, abundant in both fertilized and unfertilized eggs but rapidly degrading before MZT, are linked to cell division and protein degradation, facilitating rapid embryonic development during the first 4 h postfertilization (hpf). Notably, the Gata4 TF is an early determinant of dorsal-ventral patterning and it makes sense that it is a constituent of Cluster 3.^86^ Cluster 4 proteins, peaking during gastrulation and neurulation, are associated with cell division, translation elongation, embryonic organ development, and chromatin modification. This reflects the shift from maternal to zygotic production, high translational activity, cell division, and the onset of tissue differentiation. Clusters 5 to 8 exhibit a monotonous growth pattern during MZT, gastrulation, neurulation, and tailbud stages. In later stages, the focus shifts to energy generation, transport, metabolic processes, and tissue morphogenesis. These clusters are enriched with cofactors, coenzymes involved in metabolism, and actin filament organization, correlating with metabolic preparation for swimming tadpoles. Collectively, these analyses revealed proteome dynamics during development, mirroring various aspects of tissue differentiation and morphogenesis.

Next, we evaluated the utility of the proteome atlas as a tool to analyze the expression of tissue-specific marker genes, including those representing the major lineages/germ layers (Figure S4B). This revealed a series of staggered progression waves in protein expression across different tissue types. In line with existing literature,^98^ we observe that the onset of most tissue differentiation began with gastrulation at the 110-cell stage (epidermis, and endoderm). In the case of the notochord (Sec31b) and mesenchyme (Ci-Psl3), some markers emerge as early as the 16-cell stage, underscoring the unique aspects of *Ciona* embryogenesis where most cells are restricted to a single tissue fate by the start of gastrulation.^99^ Markers of differentiating neurons associated with the dorsal and lateral regions of the brain such as Synaptotagmin 1 (Syt),^100^ Cel3/4/5 (also known as Etr-1, Cel3.a),^101^ and Rlbp1 (also known as Cralbp)^100^ are also identified at relatively early stages of embryogenesis. For the muscle lineage, we observe multiple proteins expressed contemporaneously starting from the mid-tailbud II stage (Figure S4B).^37^ These examples highlight a developmental progression in protein expression patterns and how the proteome atlas effectively mirrors the establishment of definitive cellular phenotypes, in this case elongated muscles.

To further evaluate the utility of the proteome atlas, we explored aspects of temporal fate patterning, focusing on TFs and SMs that are critical for cell specialization during embryogenesis. The data cover approximately 40% of all annotated TFs and ∼60% of all SMs, kinases and phosphatases (Figure S4C). Principal component analysis (PCA) shows a smooth transition from one stage to the next, with the first two principal components accounting for over 80% of the proteome’s variance. A striking 'salt and pepper' pattern emerged when overlaying transcriptional regulators across the proteome’s development. The observed expression dynamics likely reflect a combination of tissue composition and protein accumulation, effectively separating early and late expression protein along a spatial developmental continuum (Figure S4D).

We also ranked protein changes across consecutive developmental stages to identify stage-specific proteins. This analysis highlights significant changes in protein abundance at three key stages: post-fertilization, the maternal-to-zygotic transition (MZT), and the onset of metamorphosis. Post-fertilization, the egg’s proteome exhibits substantial alterations of proteins involved in calcium signaling, mitochondrial function, and translation. The MZT phase shows a surge in proteins related to organogenesis. As swimming tadpoles transition toward metamorphosis there is an increase in proteins associated with tail reabsorption. Examples include the TF Hox10^102^ (Figure S4E).

### Quantitative mRNA-protein expression landscapes

Cellular protein concentrations are modulated via transcriptional and translational mechanisms.^103^ By integrating transcriptomic and proteomic data from stage-specific embryos, we can explore the extent to which RNA signatures explain protein dynamics. First, we observe that protein and transcript expression vary significantly, spanning different orders of magnitude (Figure S5A). Moreover, consistent with existing literature,^104^^,^^105^ proteins encoded by low-abundance genes are underrepresented, indicating proteome coverage is not yet exhaustive (Figure S5B). We also notice strong variations in quantitative levels at each developmental stage, evident at both the protein and gene levels. There is little overlap in the rank order or even the identity of the most abundant proteins and mRNAs at any given stage (Figure S5C).

The overall correlation between the 7,021 mRNA and protein pairs is low, with a median Pearson correlation of −0.012 (Figure 2D, Table S5), similar to previous studies (Figure S5).^4^^,^^5^^,^^6^^,^^7^ Our approach assesses how mRNA and protein pairs change over the developmental timeline rather than a snapshot of a specific stage. Figure 2E illustrates an example of TF with high Pearson correlation between RNA-protein dynamics. Additionally, Figure S6 presents a selection of TFs known to play significant roles in the early development of *Ciona*.^98^

Using k-means co-clustering of mRNA and protein pairs, we identified 5 distinct cluster dynamics (Figure 2F). We found that the genes involved in DNA replication/repair, centriole elongation/replication, rRNA processing, and protein localization to the nucleus have maternally loaded RNA and the most static protein dynamics. Metabolic processes broadly span all of the clusters, implying that metabolic processes are not categorized by a specific dynamic pattern. Axon development, heart development, and muscle filament sliding/contraction genes are expressed at the transcript and protein level during the tailbud and larval stages of development. These data suggest that the genes in the more dynamic clusters are preferentially associated with organogenesis while the genes in the less dynamic clusters tend to drive housekeeping or cell cycle functions (Figure 2G).

In summary, we profiled *Ciona*’s proteome and transcriptome across key developmental stages, resulting in an atlas of 7,021 protein-mRNA pairs, underscoring the complementary nature of mRNA and protein data in understanding cellular mechanisms. The dataset shows how mRNA and protein profiles can diverge and decouple due to translational regulation, demonstrating that transcriptional changes can be modified or overridden. This atlas, enriched with existing genomic and epigenomic data, provides a basis for further exploring RNA-protein dynamics during embryogenesis and systematically assessing adaptive expression of both RNAs and proteins.

### Conserved and divergent features of the *Ciona* and *Xenopus* proteomes

**Figure 3:**
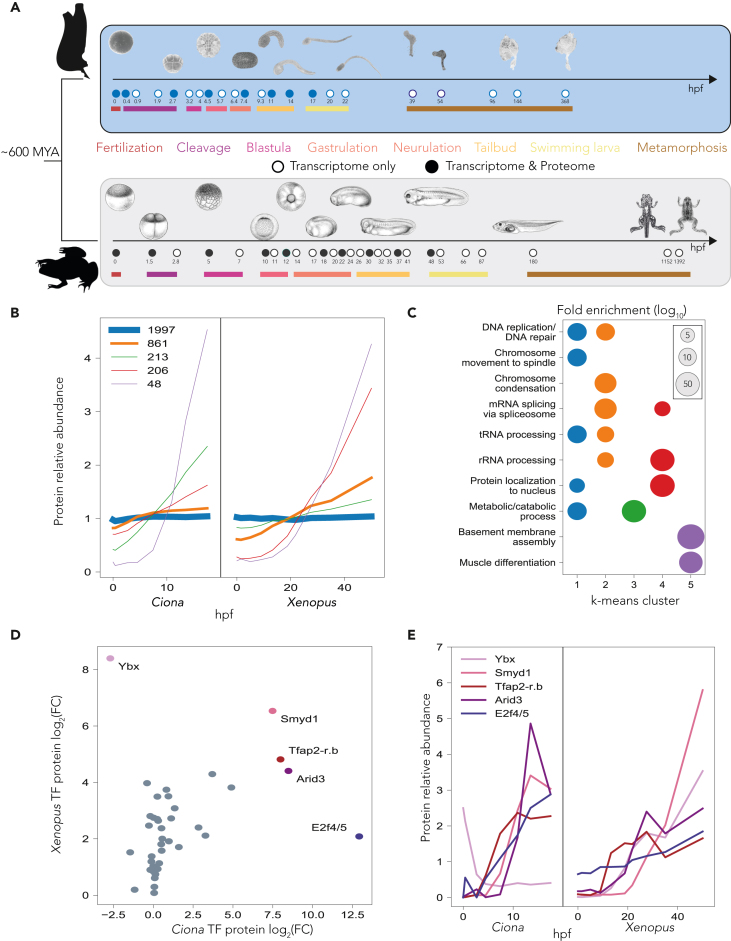
Comparison of development between chordate and vertebrate (A) Experimental design of the inter-species comparative developmental transcriptome and proteome time courses. Full circles highlight stages of development sampled for RNA-seq and proteomics. Mya, million years ago. (B) K-means co-clustering of the dynamics of orthologs (3,325) between *Ciona* and *Xenopus* development. The thickness of the line scales with the number of proteins represented in each cluster. The number of proteins in each cluster are quantified in the legend. *Xenopus* proteome time series from Sonnett et al.^106^ (C) GO term analysis identifying the functional significance of each of the clusters from B. The color of the clusters in B is kept consistent. (D) The log2 fold change (FC) protein correlation between *Ciona* and *Xenopus* TFs. Here, FC is defined as the ratio of relative protein abundance in the larva stage compared to the egg. Most TFs show similar behavior with the notable exception of Ybx. (E) Relative protein dynamics of TFs Ybx, Smyd1, Tfap2-r.b, Arid3, and E2f4/5. Each exhibit large fold changes in both organisms. Colors are preserved in these five proteins from the plotting in D. These TFs are canonically important for organism development by regulating transcriptional activation during the cell cycle, early muscle development, ectoderm development, gene activation through chromatin remodeling, and Nodal signaling respectively. Ybx exhibits signs of being maternally deposited in *Ciona*, but not in *Xenopus*, suggesting functional evolutionary divergence of this ortholog from chordate to vertebrate. *Xenopus* illustrations © Natalya Zahn (2022).

Embryogenesis progresses through distinct stages, but it remains unclear if the regulatory mechanisms guiding these transitions are conserved across species. In particular, how well are the protein dynamics of orthologues conserved over significant evolutionary distances? Is there a conservation of protein abundances in relation to the levels of their corresponding mRNAs? With these questions in mind, we compare the proteome of *Ciona* development with that of a vertebrate. We focused on the African clawed frog *Xenopus laevis*, which is very attractive for proteomics analysis^4^^,^^5^^,^^63^^,^^64^^,^^106^^,^^107^ resulting in one of the best characterized vertebrate proteomes throughout embryogenesis. *Xenopus* and *Ciona* diverged approximately 500–600 million years ago,^108^ providing a significant evolutionary distance for comparison (Figure 3A).

We applied k-means clustering to classify 3,350 one-to-one orthologous protein pairs into 5 distinct clusters, using the frog proteome time series data from Sonnet et al.^106^ (Table S7), and we identified significant similarities in proteome dynamics between these two species (Figure 3B). More than half of the shared proteins are stably expressed in both species throughout development (blue cluster, Figures 3B and 3C). This cluster is enriched for proteins involved in DNA replication, spindle formation, and chromosome movements. Clusters that capture the activity of genes involved in rRNA processing, tRNA processing, and mRNA splicing via the spliceosome show an increase in expression throughout embryogenesis in both organisms. Genes involved in metabolic and catabolic processes also shared an increase in expression throughout embryogenesis in both organisms, however with a more pronounced increase in *Ciona* (Figures 3B and 3C). Basement membrane assembly and muscle differentiation genes have similarly high expression throughout embryogenesis in both organisms (Figures 3B and 3C), including those known to have roles in late development such as Lamα5 and Smyd1.^109^^,^^110^ These results highlight the similarities of orthologous protein dynamics during the development of these highly divergent species.

We next shifted our focus to the dynamics of orthologous TFs during development. We looked at the relative expression of these proteins in swimming tadpoles over their relative expression levels in the eggs of each organism (Figure 3D). Overall, TFs that showed the most pronounced changes in *Ciona* tended to also increase their expression in *Xenopus*. Notably, Smyd1, Tfap2-r.b, and Arid3, which are known transcriptional regulators of muscle,^83^^,^^99^^,^^109^ ectoderm/neural crest development,^99^ and chromatin remodeling,^83^ respectively, exhibited similar patterns of expression in both species (Figure 3E). Importantly, we observed TFs that showed different expression dynamics between the two species. The Y-box binding protein, Ybx, exhibited inverse behavior between the two organisms. In *Ciona*, Ybx mRNA^83^ and protein are maternally deposited, whereas in *Xenopus*, it is strictly expressed after fertilization and plays a crucial role in muscle and vascular development.^111^^,^^112^ Ybx is a highly conserved protein involved in transcriptional regulation and is a component of messenger ribonucleoprotein complexes.^113^ Notably, in zebrafish, both mRNA and protein are maternally deposited and are essential for activating maternal Nodal signaling.^114^ Understanding the underlying reasons for the differential behavior of Ybx in *Ciona* and *Xenopus* requires further investigation. Despite many similarities, there are numerous differences that probably reflect species-specific functions.

We have identified conserved and unique protein dynamics across *Ciona* and *Xenopus* through comparison for more than ∼3,000 orthologous proteins. Overall, we find strikingly high conservation of protein dynamics between the two organisms even though they are separated by ∼600 million years of evolution. This analysis therefore presents an exciting opportunity to shed light on conserved regulatory processes in chordate development.

### An inverse hourglass model for proteome evolution between *Ciona* and *Xenopus*

Cross-species embryonic development is typically aligned at the transcriptome level.^48^^,^^49^^,^^50^^,^^51^^,^^53^^,^^55^ We therefore used developmental proteomes to establish stage correspondences between *Ciona* and *Xenopus* species throughout embryogenesis. We identified 7,636 one-to-one orthologs at the gene level (Tables S8 and S9).^53^^,^^115^ At the proteome level, we complemented the time series data from Sonnet et al.^106^ (comprising 3,350 one-to-one orthologs, Table S7) by using an additional independent proteome time series from Van Itallie et al.,^107^ which included 5,376 one-to-one protein pairs (Table S10).

**Figure 4:**
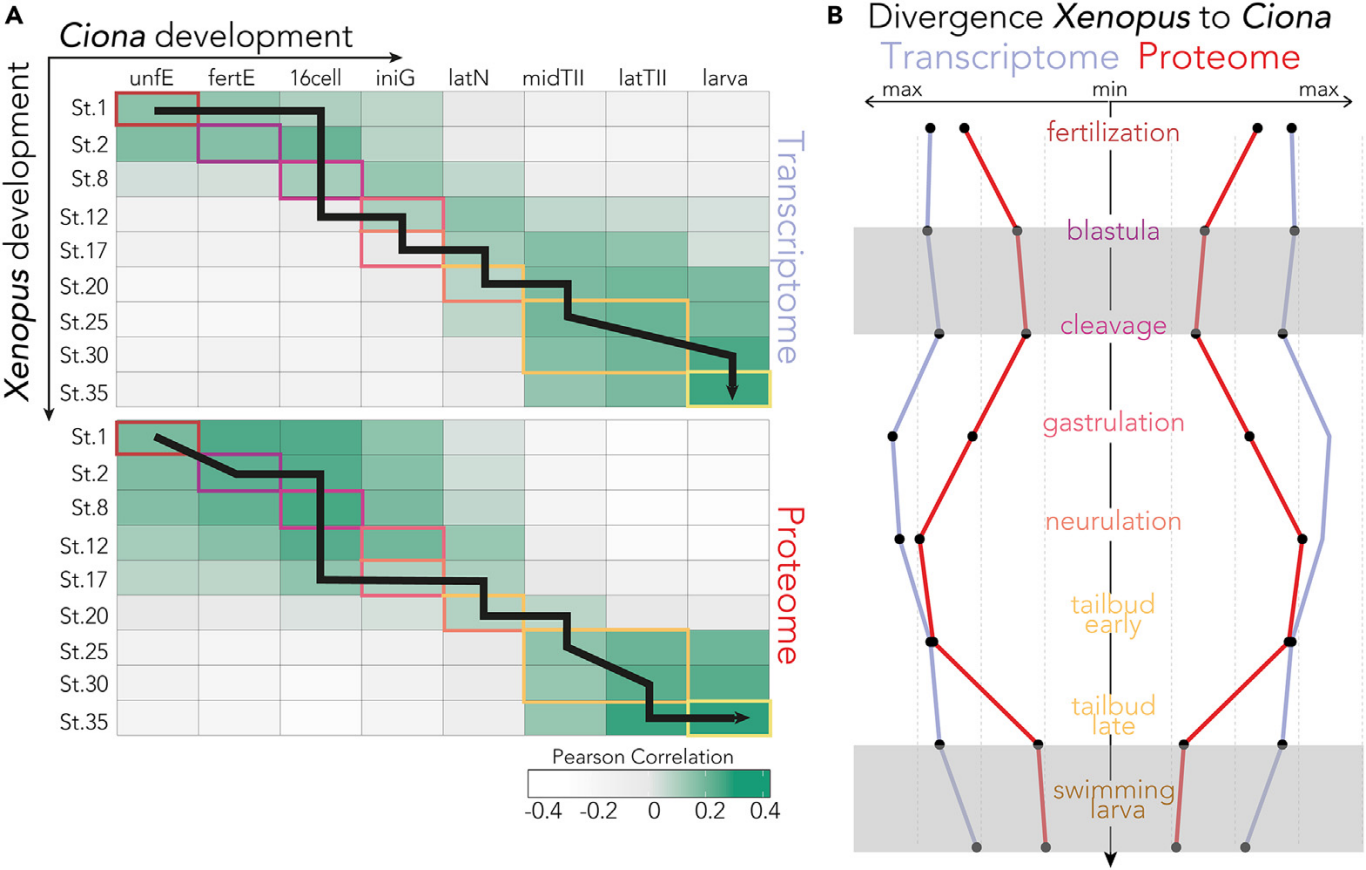
The protein anti-hourglass model (A) Similarity heatmaps showing Pearson similarity between the two species for each investigated time point. Developmental stages are color-coded as defined in Figure 3A. The black line follows the highest correlation of the *Xenopus* time-point for each *Ciona* stage (n = 3,350, *Xenopus* transcriptome from Hu et al.,^53^ and Session et al.,^115^. *Xenopus* proteome from Sonnett et al.^106^). (B) Temporal divergence of gene (blue) and protein (red) expression from *Xenopus* embryogenesis to each *Ciona* stage. Maximal similarity is represented by the smallest distance from the center line, revealing a nested hourglass model in which the proteome exhibits more evident bottlenecks at early and later stages. Gray boxes outline these periods of minimal divergence. Regardless of stage, proteins show higher similarity between the two species' developmental mapping than RNA-seq, suggesting that protein dynamics are evolutionarily more conserved than mRNA dynamics (n = 3,350, *Xenopus* transcriptome from Hu et al.,^53^ and Session et al.,^115^. *Xenopus* proteome from Sonnett et al.^106^).

Starting at the transcriptome level, we observed that 60% of the orthologs are commonly expressed in both species during the early stages, before gastrulation. This shared expression decreased to 55% during the mid-developmental transition (gastrulation and neurulation) and reached 50% in the late phase (tailbud, larva, juveniles), with the highest proportion detected in early development (Figure S7A). We next sought to determine how changes in gene expression mark different developmental stages. We found that gene expression patterns between the two species do not show abrupt changes between stages but rather change gradually and continuously throughout embryonic development. This indicates a single continuum of differentiation, rather than distinct subsets, with smooth transitions across consecutive stages. The greatest transcriptomic similarity occurs at hatching, when excluding *Ciona* metamorphosis stages (Figures 4A and S7B; Tables S8 and S9).

Comparison of the shared proteome reveals striking differences with the analysis of transcriptomes. The proteomes exhibit distinct phases of shared expression, one early and one late, which are divided by a sharp mid-developmental transition (Figure 4A). The two species showed increasing proteome divergence with each other as they undergo neurulation. This pattern is consistent with an inverse hourglass model with the highest divergence during gastrulation and neurulation (Figures 4A, S8, and S9). The early developmental phase may be subject to more functional constraints and less refractory to change, while the larval stage, crucial for forming a swimming tadpole in both species, shows overlapping protein functions and similar phenotypes.

The proteogenomic patterns revealed by this study remain consistent across various types of comparisons and are robust against different parameters used in constructing the correlation matrix (Pearson (r), Spearman (ρ), Cosine) (Figure S7C), and potential stage sampling biases (Figures S7B, S8, and S9). For example, extending the *Ciona* time series from 8 to 20 stages (from egg to juveniles, Table S8^53^ and the *Xenopus* series to 17 distinct time points (from egg to swimming and feeding tadpoles, Table S9)^53^^,^^115^ again showed maximal transcriptomic similarity at hatching (Figure S7B). Similarly, when analyzing a different proteome dataset for inter-species comparison,^106^^,^^107^ the dual-phase pattern is still evident. This *Xenopus* time series included two additional time points beyond those previously analyzed, effectively spanning the first 120 (hpf) of embryogenesis (Figures S8 and S9).

To map stage transitions in the embryonic timeline, we classified stages with similar morphological events in both species, including cleavage, blastula formation, gastrulation, neurulation, tailbud and swimming larva. We determined the highest correlation points for each stage using both transcriptome and proteome data. By connecting these points (shown as a black line in Figure 4A), we assessed whether mRNA or protein expression better matched the known phenotypic stages. This analysis revealed that protein correlations more closely followed the established mapping of equivalent developmental stages (Figure 4A), indicating that proteomes provide a more accurate representation of embryonic stages compared to transcriptomes (Figure 4A).

Our results are consistent with an inverse hourglass model for protein conservation whereby protein activity is most divergent at mid-developmental stages and the molecular components that comprise early and late embryogenesis are more conserved (Figures 4B, S8, and S9). We hypothesize that this divergence might represent the distinct mechanisms of gastrulation and neurulation in the two species. In *Ciona*, gastrulation takes place via a cup-shaped gastrula driven by invagination of the endoderm, whereas in *Xenopus*, convergent extension of mesoderm and epidermal epiboly play important roles. Most importantly, *Ciona* differs temporally from its vertebrate cousin by specifying its axis at the neurula stage, rather than at gastrulation.^116^ In frog development, Stage 9 signifies the beginning of gastrulation. Maternal deposits and translation play a significant role in shaping early embryogenesis. It is likely that similar proteins and pathways are conserved across species for timing and initiating this crucial phase, as evidenced by the high conservation observed in the proteome during this period. However, as gastrulation begins, the dynamics of embryogenesis shift, the mechanisms underlying this process start to differ significantly among species, setting the stage for the zygotic genome to take over gradually. This divergence is reflected in low or negligible signals of conservation observed in the blastula stage transcriptome among different species. New genes need to be expressed becoming more diverse and species-specific to evolutionary adaptations. The highest similarity between the species proteomes is observed at the larval stage, likely due to shared structural and ecological needs of swimming larvae.

Throughout all stages, we noticed that the proteome correlations were always higher than the transcriptome correlations (Figures 4A and 4B). This suggests that protein behavior is more evolutionarily conserved over time than mRNA behavior, likely because proteins are directly responsible for carrying out functions.^61^^,^^117^ It is possible that post-transcriptional mechanisms, such as variations in translation or protein degradation rates, have evolved to offset differences in mRNA dynamics.

The proteome closely reflects an organism’s physical traits, offering a more accurate measure of developmental and evolutionary differences within chordates. This underscores the importance of proteomics for evolutionary studies across species. However, previous gene ontology analysis linked variations in the transcriptome to specific biological functions. Regulatory mechanisms, including post-transcriptional, translational, and protein-degradation processes, appear to compensate for mRNA levels dissimilarity, aligning protein abundances with evolutionarily preferred levels.^61^^,^^118^^,^^119^ This suggests a synergy between genetic drift and regulatory mechanisms in chordate evolution, focusing on key regulatory genes essential for developmental processes and post-translational regulation. Our study highlights the significance of the simple chordate *Ciona* in understanding chordate development, proving its worth as a model for future comparative research, particularly in studying proteome stability and its evolutionary implications.

### Limitations of the study

Our analysis is subject to certain limitations. The proteome atlas identifies ∼15,000 expressed genes and ∼7,000 proteins. Nearly 40% of the proteome remains uncharacterized, likely missing proteins expressed during later stages, such as metamorphosis, which our embryo-centric analysis does not cover. It is also possible that a number of RNAs and proteins are exclusively expressed in juveniles or adults, representing another gap yet to be addressed. Additionally, the detection of certain proteins is challenged by their incompatibility with standard proteomics methods, including precipitation and digestion steps, or due to their low abundance.^13^^,^^120^ Our analysis, based on whole embryos, inherently reflects average protein levels across diverse cell types. Our study includes the analysis of different stages of *Ciona* embryogenesis, however we would like to point out that there is a comparative under-representation of metamorphosis and juvenile stages.

## STAR★Methods

### Key resources table

**Table:** 

REAGENT or RESOURCE	SOURCE	IDENTIFIER
**Biological samples**		

*Ciona robusta* formerly *Ciona intestinalis* type A	San Diego, USA	N/A

**Chemicals, peptides, and recombinant proteins**		

Pierce Protease Inhibitor Mini Tablets, EDTA Free	Thermo Scientific	Cat#PI88666
Lysyl Endopeptidase, MS Grade (Lys-C)	Wako Pure Chemical	Cat#125-05061
Sequencing Grade Modified Trypsin	Promega	Cat#V5111
RNase A, DNase and protease-free	Thermo Scientific	Cat#EN0531
Trypsin Protease, MS Grade	Thermo Scientific	Cat#90305
TRI Reagent	Sigma-Aldrich	Cat#93289
TMTsixplex Isobaric Label Reagent Set	Thermo Scientific	Cat#90062
Sep-Pak C18 1 cc Vac Cartridge	Waters	Cat#WAT054955
Pierce C18 Spin Tips & Columns	Thermo Scientific	Cat#84850
TURBO DNase	Invitrogen	Cat#AM2238

**Critical commercial assays**		

Quick Start Bradford Protein Assay Kit 1	Bio-Rad	Cat#5000201
Proteomics Dynamic Range Standard Set	Sigma-Aldrich	Cat#232-650-8
RNA Clean & Concentrator Kit	Zymo	Cat#R1017
PrepX RNA-Seq for Illumina Library Kit	Takara Bio	Cat#640097
Pierce BCA Protein Assay Kits	Thermo Scientific	Cat#23225

**Deposited data**		

Raw and analyzed RNA-seq data	This paper	GEO: GSE237005
Raw proteomics data	This paper	ProteomeXchange: PXD043619
*Ciona* bulk RNA-seq	Reeves et al.^39^; Kaplan et al.^67^; Sharma et al.^68^; Wang et al.^69^	NCBI SRA PRJNA376667,PRJNA508201,PRJNA498494,PRJNA529900
KH *Ciona* Transcriptome	ANISEED^121^	https://aniseed.fr/
*Homo sapiens* proteome	Uniprot^70^	Proteome ID: UP000005640
*Gallus gallus* proteome	Uniprot^70^	Proteome ID: UP000000539
*Xenopus tropicalis* proteome	Uniprot^70^	Proteome ID: UP000008143
*Danio rerio* proteome	Uniprot^70^	Proteome ID: UP000000437
*Branchiostoma floridae* proteome	Uniprot^70^	Proteome ID: UP000001554
*Strongylocentrotus purpuratus* proteome	Uniprot^70^	Proteome ID: UP000007110
*Ciona robusta* proteome	Uniprot^70^	Proteome ID: UP000008144
KY21 *Ciona* proteome	Satou et al.^72^	http://ghost.zool.kyoto-u.ac.jp/download_ht.html
*Xenopus laevis* v10.1 proteome	NCBI	NCBI RefSeq assembly GCF_017654675.1
*Ciona* time-series RNA-seq data	Hu et al.^53^	NCBI SRA PRJDB3785
*Xenopus laevis* time-series RNA-seq data	Hu et al.^53^; Session et al.^115^	NCBI SRA PRJDB3785, PRJNA296953
*Xenopus laevis* time-series proteomics data	Sonnett et al.^106^	ProteomeXchange: PXD007915
UPS2 proteomics standards FASTA file	Sigma-Aldrich	https://www.sigmaaldrich.com/deepweb/assets/sigmaaldrich/marketing/global/fasta-files/ups1-ups2-sequences.fasta

**Software and algorithms**		

Mass Spec Protein Reference Tool	Wühr et al.^64^	https://kirschner.med.harvard.edu/tools/mz_ref_db.html
Python	Python Software Foundation	https://www.python.org
BLAST (version 2.10.1)	Altschul et al.^122^	https://blast.ncbi.nlm.nih.gov/doc/blast-help/downloadblastdata.html; RRID:SCR_001653; RRID:SCR_001010
Trinity (version 2.11)	Grabherr et al.^123^	https://github.com/trinityrnaseq/trinityrnaseq/releases; RRID:SCR_013048
SeqClean	Dana-Farber Cancer Institute	https://sourceforge.net/projects/seqclean/files/
RepeatMasker (version 4.1)	Smit et al.^124^	https://www.repeatmasker.org/RepeatMasker/; RRID:SCR_012954
TGICL (version 2.1)	Pertea et al.^125^	https://sourceforge.net/projects/tgicl/files/tgicl%20v2.1/
CAP3	Huang et al.^126^	https://faculty.sites.iastate.edu/xqhuang/cap3-assembly-program; RRID:SCR_007250
CD-HIT (version 4.8.1)	Fu et al.^127^; Li et al.^128^	https://github.com/weizhongli/cdhit; RRID:SCR_007105
R (gProfiler, topGo)	Kolberg et al.^129^; Alexa et al.^130^	RRID:SCR_006809; RRID:SCR_014798
FastQC (version 0.12.0)	Babraham Bioinformatics	https://github.com/s-andrews/FastQC; RRID:SCR_014583
Trimgalore (version 0.6.10)	Babraham Institute	https://github.com/FelixKrueger/TrimGalore; RRID:SCR_011847
Salmon	Patro et al.^131^	https://github.com/COMBINE-lab/salmon; RRID:SCR_017036

**Other**		

Genome annotation files, transcription factor and signaling molecules databases used for RNA-seq and proteomics analyses, alignment files used in orthology assignment and other additional files	This paper	https://github.com/andreamariossi/proteome_ciona
Adapted *Ciona* schematics	Hotta et al.^132^	https://chordate.bpni.bio.keio.ac.jp/chordate/faba/1.4/top.html
Xenopus illustrations	Xenbase, Zahn et al.^133^	https://www.xenbase.org/xenbase/zahn.do

### Resource availability

#### Lead contact

Further information and requests should be directed to the lead contact, Martin Wühr (wuhr@princeton.edu).

#### Materials availability

Materials generated for this study are available on request from Martin Wühr (wuhr@princeton.edu).

#### Data and code availability

• Data: The raw data associated with the RNA-seq experiments and gene expression matrices are available in GEO under the accession number: GSE237005. The mass spectrometry experiments presented in this study have been deposited to the ProteomeXchange Consortium (http://www.proteomexchange.org/). Embryo developmental proteome (deposited via the PRIDE partner repository) with accession number: PXD043619. Genome annotation files, transcription factor and signaling molecules databases used for RNA-seq and proteomics analyses, alignment files used in orthology assignment, and additional files are publicly available on GitHub (https://github.com/andreamariossi/proteome_ciona).
• Code: All code to reproduce this study is publicly available on GitHub (https://github.com/andreamariossi/proteome_ciona).
• Other items: Additional information required to reanalyze the data reported in this paper is available from the lead contact upon request.

### Experimental model and study participant details

#### *Ciona* handling and embryos collection

Wild type adult hermaphrodite *Ciona robusta* (formerly known as *Ciona intestinalis* Type A)^134^ were obtained from M-Rep located in San Diego, CA and maintained in artificial seawater (Instant Ocean) at 18°C, under continuous illumination. Dechorionation and *in vitro* fertilization procedures were conducted following the protocol described in.^135^ For each time point in the time series, embryos were staged and collected according to^132^ at approximately 18°C and a total of 150 embryos were placed in Trizol for RNA extraction, while approximately 3,000 embryos were rapidly frozen in liquid nitrogen for protein TMTproC sample preparation. All samples were then stored at −80°C until further use. For absolute mass spectrometry analysis, approximately 5,000 unfertilized dechorionated eggs were directly snap-frozen.

### Method details

#### SNP prevalence between *ciona* batches

One concern is the presence of single nucleotide polymorphisms (SNPs), a characteristic feature of ascidian evolution,^73^^,^^136^ which can cause protein sequence polymorphisms and lead to incorrect peptide inference during the processing of MS data. We evaluated the potential influence of SNPs on peptide quantification accuracy. We obtained bulk RNA-seq data from two batches of 16-cell *Ciona* embryos. Each batch was assembled via Trinity, then translated into protein reference databases with the mass spec protein reference tool (https://kirschner.med.harvard.edu/tools/mz_ref_db.html).^64^ We reciprocally BLASTed each database against the other and found 16,037 shared proteins. These shared proteins were trypsin digested *in silico*. 98.8% of the resulting peptides were identical between these batches while only 1.2% were wholly unique to one batch or the other indicating minimal influence of intra-specific genetic variability on peptide recognition.

#### Generating protein reference database

The protein reference database, a FASTA file containing all potential proteins from the species under study, was used to generate *in silico* tryptic peptides and reference MS/MS spectra for peptide identification. 1,222,451,669 *Ciona* bulk RNA-seq reads from numerous studies^39^^,^^67^^,^^68^^,^^69^ were assembled *de novo* via Trinity (version 2.11) into 2,328,005 transcripts.^123^ The 55,974 transcripts making up the KH *Ciona* transcriptome (KHNCBI.Transcript.2018.fasta, retrieved from ANISEED)^121^ were integrated alongside our *de novo* transcripts. The transcripts were cleaned and trimmed via SeqClean (http://compbio.dfci.harvard.edu/tgi/software/), then masked for common repeat motifs via RepeatMasker (version 4.1).^124^ The masked transcripts were clustered via TGICL (version 2.1) and assembled via CAP3.^125^^,^^126^ The resulting contigs and singletons were searched against a database of model organism containing human (*Homo sapiens*), red junglefowl (*Gallus gallus*), western clawed frog (*Xenopus tropicalis*), zebrafish (*Danio rerio*), florida lancelet (*Branchiostoma floridae*), pacific purple sea urchin (*Strongylocentrotus purpuratus*), and urochordate (*Ciona robusta*) using BLASTX (version 2.10.1).^122^ The BLASTX report was parsed and the transcripts were translated into proteins. The translated proteins were processed to remove redundancies with a CD-HIT (version 4.8.1) threshold of 95%.^127^^,^^128^

#### Proteomics sample preparation

Samples were prepared by lysing frozen embryos in lysis buffer (50 mM HEPES pH 7.2, 2% SDS, and 1x protease in artificial saltwater) followed by clarification via centrifugation. Lysates were diluted to 2 μg/μL with 100 mM HEPES (pH 7.2). DTT was added to a concentration of 5 mM and samples incubated for 20 min at 60°C. After cooling to room temperature (RT), N-ethylmaleimide (NEM) was added to a concentration of 20 mM and samples incubated for 20 min at RT. 10 mM DTT was added and samples incubated for 10 min at RT to quench NEM. 200 μL of each sample were brought up to 2 mL with 800 μL MeOH, 400 μL chloroform, and 600 μL water. Samples were centrifuged at 20,000 g for 2 min at RT. Upper layer was discarded and 600 μL MeOH was added. Samples were centrifuged at 20,000 g for 2 min at RT. Supernatant was discarded and 500 μL MeOH was added.^137^ Samples were centrifuged at 20,000 g for 2 min at RT. Supernatant was discarded and the pellet was air dried. Pellet was resuspended in 6 M GuaCl, 10 mM EPPS pH 8.5 to ∼5 mg/mL.

For the label-free samples, UPS2 standards (Sigma-Aldrich) were added to a final concentration of 27 ng/μL in the 450 μg protein samples. Samples were diluted with 10 mM EPPS pH 8.5 to 2 M guanidine hydrochloride. Samples were digested overnight at RT in LysC (Wako) at a concentration of 20 ng/μL. Samples were further diluted with 10 mM EPPS pH 8.5 to 0.5 M guanidine hydrochloride. 20 ng/μL LysC and 10 ng/μL trypsin (Promega) were added to each sample and incubated for 16 h at 37°C. Peptide supernatant was cleared by ultracentrifugation at 100,000 g for 1 h at 4°C (Beckman Coulter, 343775), then vacuum-dried overnight.

For TMTpro-labeling, samples were digested with LysC and trypsin as above, then resuspended in 200 mM EPPS pH 8.0. pre-mixed TMTpro tags (8-plex Thermo Fisher Scientific 20 μg/μL in dry acetonitrile stored at −80°C) at a 5 μg TMTpro: 1 μg peptide ratio. To cover the eight developmental time series samples, tags are as follows: 126 - unfertilized egg; 128C – fertilized egg; 129N–16-cell; 130C–initial gastrula; 131N – late neurula; 131C – mid tailbud II; 133C – late tailbud II; 134N – larva. Samples were incubated for 2 h at RT. Reactions were quenched by addition of hydroxylamine (Sigma, HPLC grade) to a final concentration of 0.5% for 30 min at RT. Samples were pooled into a single tube, cleared by ultracentrifugation at 100,000 g for 1 h at 4°C (Beckman Coulter, 343775), then and vacuum-dried overnight.

For either label-free or TMTpro-labeled, samples were resuspended with 10 mM ammonium bicarbonate (pH 8.0) with 5% acetonitrile to 1 μg/μL. Samples were separated by medium pH reverse phase HPLC (Zorbax 300Extend C18, 4.6 × 250 mm column) into 96 fractions.^14^^,^^138^ The fractions were then pooled into 24 fractions,^139^ dried, and resuspended in HPLC grade water. Samples were then desalted via homemade stage tips with C18 material (Empore) and resuspended to 1 μg/μL in 1% formic acid.^140^

#### Drawings

*Ciona* schematics are adapted from FABA (FABA Four-dimensional Ascidian Body Atlas)^132^ and *Xenopus* illustrations from Xenbase (www.xenbase.org RRID:SCR_003280) and Natalya Zahn.^133^ Source icons with BioRender.com.

### Quantification and statistical analysis

#### Proteomics analysis

Approximately 1 μg per sample was analyzed by LC-MS, as previously described.^138^ LC-MS experiments were analyzed on an nLC-1200 HPLC (Thermo Fisher Scientific) coupled to an Orbitrap Fusion Lumos MS (Thermo Fisher Scientific). Peptides were separated on an Aurora Series emitter column (25 cm × 75 μm ID, 1.6 μm C18) (Ionopticks), held at 60°C during separation by an in-house built column oven. Separation was achieved by applying a 12%–35% acetonitrile gradient in 0.125% formic acid and 2% DMSO over 90 min for fractionated samples. Electrospray ionization was enabled by applying a voltage of 2.6 kV through a MicroTee at the inlet of the microcapillary column. For the label-free samples, we used the Orbitrap Fusion Lumos with the label-free method with data-dependent acquisition (DDA) previously described.^64^ For the TMTpro samples, we used the Orbitrap Fusion Lumos with the TMTproC method previously described.^14^

Mass spectrometry data analysis was performed essentially as previously described^106^ with the following modifications. The raw MS files were analyzed using the GFY software licensed through Harvard University. MS2 spectra assignment was performed using the Sequest algorithm^141^ by searching the data against either our reference protein dataset described above, the KY21 *Ciona* proteome,^141^ or the Uniprot *Ciona* proteome.^70^

For label-free analysis, these proteomes were merged with the UPS2 proteomics standards FASTA file (Sigma-Aldrich) along with common contaminants. Peptides that matched multiple proteins were assigned to the proteins with the greatest number of unique peptides. To control for peptide false discovery rate, target-decoy search strategy was used where reverse sequences were searched in parallel with forward sequences.^94^ Filtering was performed using a linear discriminant analysis (LDA) that accounts for parameters from Sequest’s database search output, such as XCorr, deltaCorr, missed cleavages, charge state, peptide length, and the fraction of matched ions was also implemented to distinguish genuine peptide spectral matches (PSMs) from reverse hits. The data were then filtered to 0.5% FDR on the peptide level and 1% FDR on the protein level.^95^^,^^142^

#### Absolute protein concentration estimates in unfertilized egg

Protein concentration in the label-free egg sample was calculated by building a standard curve of MS signal to UPS2 standard concentration. The UPS2 known standard concentrations were obtained from Sigma Aldrich and concentrations were converted to log space. The MS signal area was also converted to log space and Thiel regression was performed to obtain a standard curve. Signal area was then converted to concentration and scaled to a total protein concentration of 2 mM. A cutoff of 0.01 μM was applied for low concentration protein. Information on known protein complexes was obtained from the CORUM Protein Complexes dataset.^89^ A two-way ANOVA, followed by a post-hoc Tukey HSD test, was applied to assess the distribution of protein concentrations.

#### Proteomics data processing

GFY output tables for TMTcPro MS were filtered for human protein contaminants, reversed sequences and proteins which were only identified based on modified peptides as previously described.^14^

Annotations and classifications of transcription factors, signaling molecules, kinases, and phosphatases are based on data merged from the Ghost website^143^ and.^121^^,^^144^ The proportional coverage of these families within our dataset was determined by counting the number of members that could be identified at the protein level.

K-means clustering was performed using the kmeans function in R with nstart = 100. The number of clusters was selected to 8 to capture overall protein dynamics. Further cluster increases did not reveal new cluster dynamics. GO enrichment analyses were used to assign categories to each cluster using gProfiler.^129^

Principal component analysis (PCA) was performed in R with prcomp function from the stats package. Annotations for families of transcription factors, signaling molecules, kinases, and phosphatases were then overlaid on the graphs.

For the calculation of cumulative abundance, proteins and genes were initially ranked from highest to lowest. The total expressed as a percentage is plotted against their rank order. The names or identifiers of the seven most abundant transcripts or proteins (rank 1 to 7) are listed in descending order for the respective stage.

To measure the similarity between the proteome and transcriptome datasets, Pearson’s correlation coefficient (r), Spearman’s rank correlation coefficient (ρ), and Cosine distance were calculated for each individual gene-protein pair across all stages. These coefficients were then plotted as histogram distributions.

#### *Ciona* and *Xenopus* protein orthologs

Reciprocal protein-protein BLAST (RHB) (BLASTP, version 2.10.1) was used to identify orthologs between *Ciona* and *Xenopus*.^122^ *Ciona* and *Xenopus* alternated as query and reference. For each BLASTP, the max target sequence was set to 1, e-value threshold was set to 0.01, and the matrix set to BLOSUM45. The query ID, reference ID, e-value, and bit score were logged for each match. “best-match” protein orthologs between *Ciona* and *Xenopus* based on the criteria of (1) lowest e-value and (2) highest bit score. Only proteins confirmed in both directions as “best-match” were used in the cross-species proteomic analysis (Table S6).

#### Comparative proteomics

The extent of conservation or divergence in protein expression among chordates and vertebrates was assessed by comparing the proteome of *Xenopus laevis* with that of *Ciona*. Two independent frog time series were reanalyzed: one comprising 8 time points from Sonnet et al.^106^ (Table S7) and another with 10 time points from Itallie et al.^107^ (Table S10). These series collectively cover frog embryogenesis comprehensively, overlapping at three stages (St1, St12, and St30). All three datasets were first subjected to median-based normalization. Then, the dynamics of proteins in each dataset were scaled to sum to 1 across the time series, allowing for comparison of expression across species. Correlation coefficients, including Pearson (r), and Spearman (ρ), were calculated using pairs of orthologs. These orthologs were identified based on RHB methods as explained earlier, for all pairwise combinations of developmental stages. To co-cluster the *Ciona*-*Xenopus* proteomes across developmental stages (data from Sonnet et al.^106^), we used k-means clustering. Each cluster was then assigned to a functional category, based on its overall gene expression and GO enrichment profiles.

#### RNA sequencing

For each of the eight embryonic stages, a total of 150 embryos were collected and stored at −80°C in Trizol (Thermo Fisher Scientific). We prepared two biological replicates, one replicate consisted of embryos from the same *in vitro* fertilization batch for proteomic analysis. The other replicate was collected from an independent developmental time course. Total RNA was isolated using the Clean and Concentrator Zymo kit (Zymo), with genomic DNA (gDNA) removal achieved through on-column treatment with Turbo DNase (Invitrogen) at room temperature for 10 min. The resulting RNA was re-suspended in 15 μL of DEPC-treated water and quantified using a NanodropTM and Qubit (Thermo Fisher Scientific), while its quality was assessed using a Bioanalyzer 2100 (Agilent Technologies). The RNA integrity number (RIN) values ranged between 8 and 10. cDNA libraries were prepared using the PrepX RNA-seq directional protocol (Takara Bio) following the manufacturer’s instructions and utilizing an Apollo 324 robot. For mRNA enrichment and separation from rRNA, the oligo dT-based mRNA isolation kit (Takara Bio) was employed. The libraries were sequenced on the NovaSeq platform (Illumina) at the Genomics Core Facility at Princeton University with a depth of 20–40 million paired-end strand-specific reads.

Quality assessment of raw and trimmed 61-bp paired reads was performed with FastQC (version 0.12.0). Trimgalore (version 0.6.10) was used to trim the raw RNA-seq reads, removing adapters and primer contamination and poor quality base call (Q < 25). Reads shorter than 30 nt after trimming were discarded. The trimmed RNA-seq reads were then mapped to the KY21 transcriptome using Salmon (v0.42.4, with parameters --libType A, --seqBias, --gcBias, --validateMappings).^131^ Details about alignment quality are given in Table S4. mRNA quantities are presented as transcripts per million (TPM), with a cutoff of 2 TPM as the lower limit for detection across all samples. This cutoff was determined based on the inspection of distribution density plot and corroborated by known markers visualized from *in situ* hybridization chain reaction (HCR) studies^145^ at the 16-cell stage, which is when the newly zygotic genes are activated. For each stage, RNA data from biologically independent experiments were pooled to estimate average gene expression.

For *Ciona*, the extended time-series RNA-seq data was obtained from Hu et al.^53^ (Table S8). For *Xenopus leavis*, data was sourced from Session et al.^115^ and Hu et al.^53^ (Table S9). Gene expression for each species was estimated using Salmon,^131^ with KY21 annotation for *Ciona* and *Xenbase* X. laevis v10.1 annotation for frog. Gene-level expression was obtained by summing up TPMs from all transcript isoforms per gene using tximport R package.^146^ For each stage, RNA data from biologically independent experiments were pooled to estimate average gene expression. A gene was considered expressed if it had a TPM ≥2.

To compare gene expression across embryonic stages between the two species, we utilized orthologs, as identified by reciprocal best hits (RBHs). To normalize the data for distinct expression levels and mitigate the impact of highly expressed genes, we applied quantile normalization using the preprocessCore package from Bioconductor.^147^ We employed several metrics to estimate gene expression divergence between the two species, including Pearson (r) and Spearman (ρ) correlations, and Cosine similarity.

#### Gene set enrichment analysis

Gene Ontology (GO) term enrichment analyses were conducted using the gProfiler and topGo functional annotation tools.^129^^,^^130^ For each cluster, genes were analyzed against a background list comprising all genes expressed across all time points. Enriched GO terms were identified in the categories of ‘molecular function’, ‘cellular component’, and ‘biological process’. A Benjamini-corrected P-value threshold of 0.01 was applied to determine significant enrichment.

## Supplemental information

Document S1. Figures S1–S9 and Table S4

Table S1. Ciona absolute protein abundance in unfertilized egg, related to Figure 1

Table S2. Ciona relative protein abundance time series, related to Figure 2

Table S3. Ciona TPM RNA-seq time series, related to Figure 2

Table S5. Ciona relative mRNA-protein dynamics, related to Figure 2

Table S6. Ciona-Xenopus one-to-one orthologs, related to Figure 3

Table S7. Ciona-Xenopus protein dynamics from Sonnett et al. 2018, related to Figure 3

Table S8. Ciona TPM RNA-seq time series from Hu et al. 2017, related to Figure 4

Table S9. Xenopus TPM RNA-seq time series from Session et al. 2016 and Hu et al. 2017, related to Figure 4

Table S10. Ciona-Xenopus protein dynamics from Itallie et al. 2021, related to Figure 4
