# Supplementary material for: Quantitative proteome dynamics across embryogenesis in a model chordate
Source: iScience. 2024 Feb 29;27(4):109355. doi: 10.1016/j.isci.2024.109355 (PMC10951915; doi:10.1016/j.isci.2024.109355)
Supplement: Document S1. Figures S1–S9 and Table S4 [file mmc1.pdf]

iScience, Volume *27*

Supplemental information

Quantitative proteome dynamics across embryogenesis in a model chordate

Alexander N. Frese, Andrea Mariossi, Michael S. Levine, and Martin Wühr

Supplemental figures

Figure S1 LF-MS quality control and and genome-free protein reference database, related to Figure 1
Figure S2 Characterization of the identified *Ciona* proteome, related to Figure 2
Figure S3 Quality control and sample correlation of stage-specific RNA-seq samples, related to Figure 2
Figure S4 Protein expression dynamics during embryogenesis, related to Figure 2
Figure S5 Proteogenomics and dynamic range of transcript and protein expression, related to Figure 2
Figure S6 Different RNA and protein dynamics during development for TFs with known involvement in early *Ciona* development, related to Figure 2
Figure S7 Extended transcriptome analysis, related to Figure 4
Figure S8 Extended proteome analysis, related to Figure 4
Figure S9 Chordates share minimal proteome similarity during mid-developmental stages, related to Figure 4

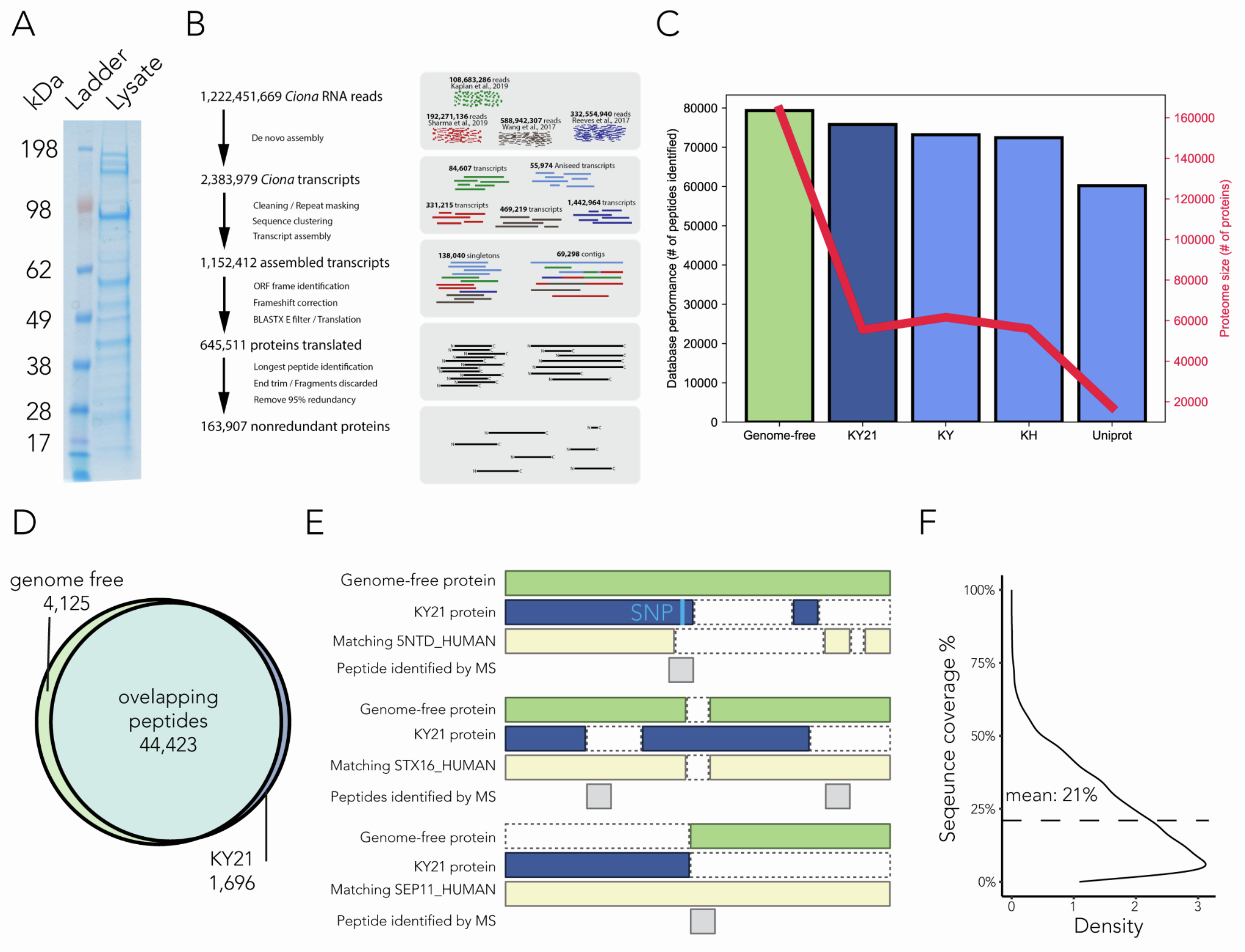

Figure S1 LF-MS quality control and and genome-free protein reference database, related to Figure 1

A, Coomassie gel of *Ciona* egg lysate. A strong band corresponding to the 100 kDa apolipoprotein-B-like yolk protein (Vitellogenin) is visible [80].

B, Bioinformatics pipeline for MS reference dataset construction. 1,222,451,669 RNA-seq reads were aggregated from five different studies and translated into a protein reference database as described previously [115] to obtain 163,907 95% non-redundant proteins.

C, The genome-free reference database, and several *Ciona* proteomes, were used to analyze the same TMTproC MS dataset. The bar heights correspond to the total number of peptides identified in the MS dataset for each annotation. The red line represents the size of the proteome database. Our reference database outperforms the KY21 proteome slightly in terms of peptide identification, but at the expense of a much larger size and manual annotation of proteins.

D, Comparison of peptides identification using genome-free reference database and KY21 proteome reference. The Venn diagram illustrates the shared and unique peptides identified using each reference.

E, Examples where genome-free reference can help improve current KY21 assembly from SNPs detection (blue insert), correction of mis-annotated coding sequences, and accurate annotation of selenoproteins.

F, Density plot of the distribution of the amino-acid based sequence coverage of the 6,219 protein detected before collapsing isoforms. The mean sequence coverage is indicated by the black dotted line.

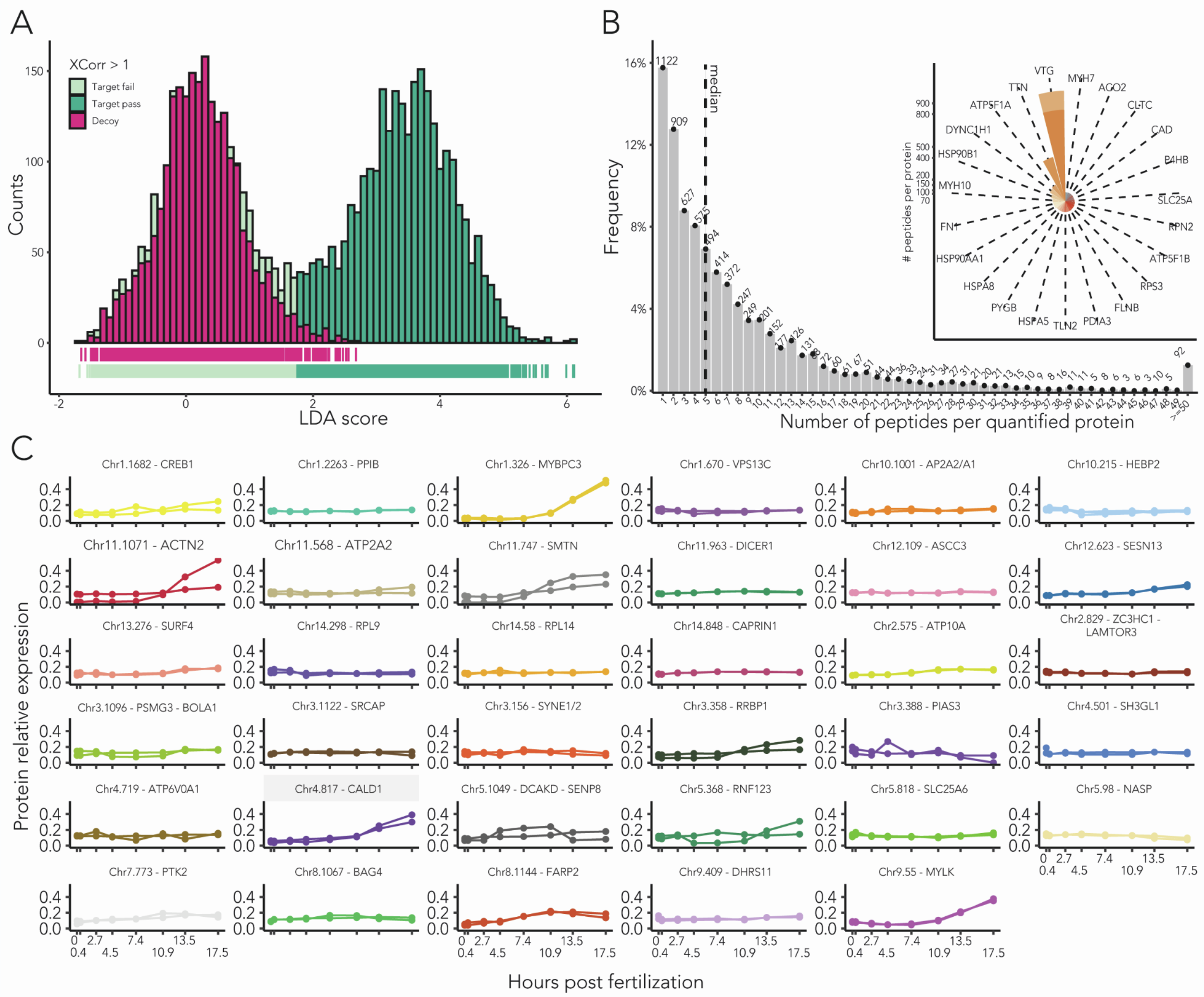

Figure S2 Characterization of the identified *Ciona* proteome, related to Figure 2

A, Linear discriminant analysis (LDA) is used to discriminate target and decoy peptides. Example of MS fraction with separation between the decoy (red) and target groups (green). 0.5 % peptide-level false discovery rate (FDR) is then estimated to discriminate between correct (dark green) and incorrect (light green) peptide−spectrum matches (PSMs). Rug plots at the bottom showing all sampling data.

B,Histogram of peptide frequencies identified per protein in the dataset, with the protein count annotated on top of each bar. Dashed line indicates median values for the dataset. Insert: number of times a peptide occurs in the top proteins.

C, Temporal expression of 35 protein isoforms represented by 2 to 4 unique splice variants.

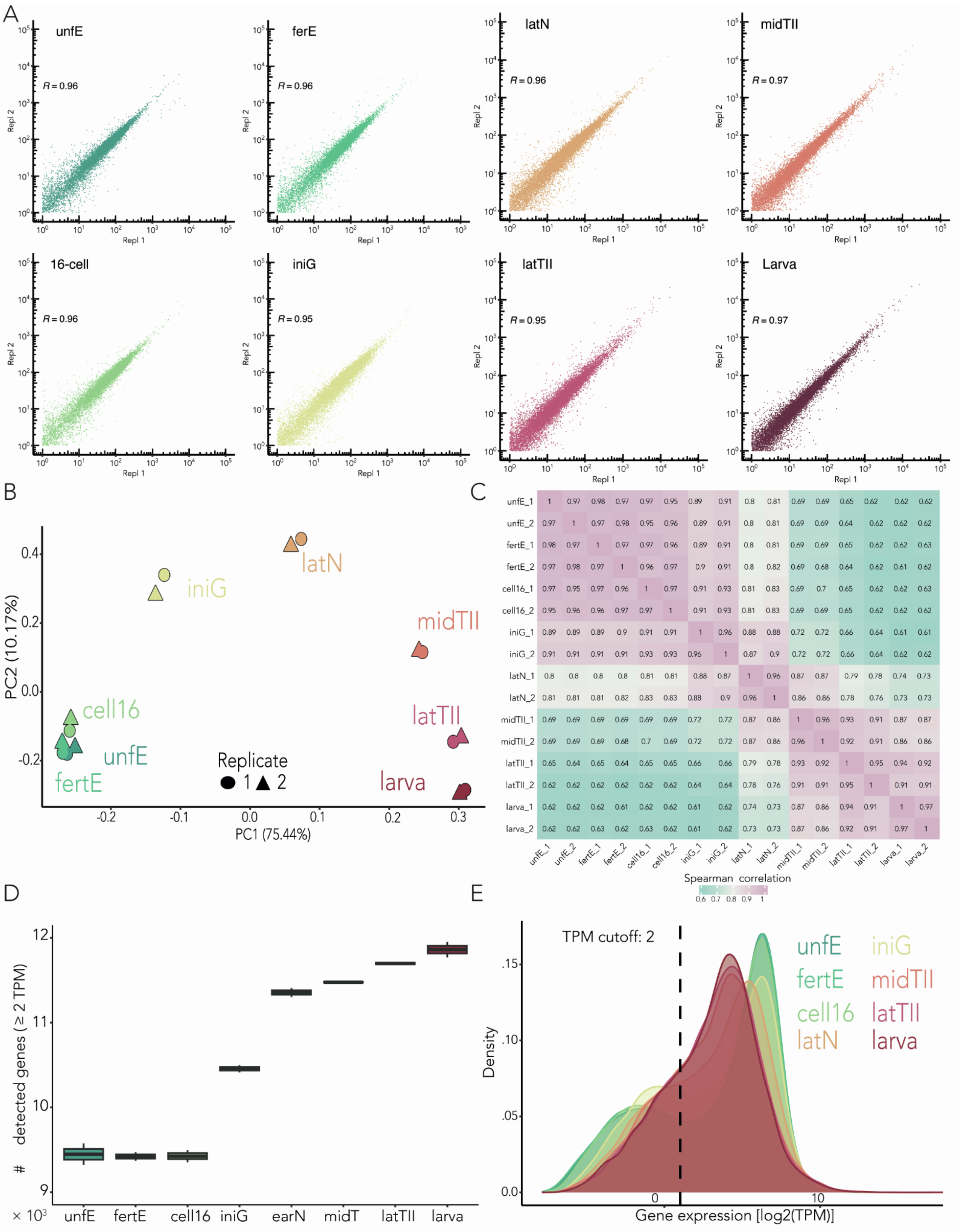

Figure S3 Quality control and sample correlation of stage-specific RNA-seq samples, related to Figure 2

A, Pairwise scatterplots of gene expression levels in transcripts per million (TPM) between biological replicates R: Pearson correlation coefficient. Stages are coloured-coded in all plots, as shown in Figure 2A.
B, Principal component analysis (PCA) of the developmental RNA-seq time course showing the first two principal components (PCs), which together explain ~85% of the variance in the data. Samples from the same stage cluster together and there is a smooth progression through developmental time.
C, Correlation matrices between RNA-seq experiments, calculated using Spearman (ρ). High agreement between biological replicates is observed.
D, Boxplot of the distributions of numbers of genes detected at each stage (≥ 2TPM).
E, Ridgeline plots of the distribution of genes by gene expression levels used to experimentally define a cut-off value of TPM ≥ 2 to deem a gene expressed.

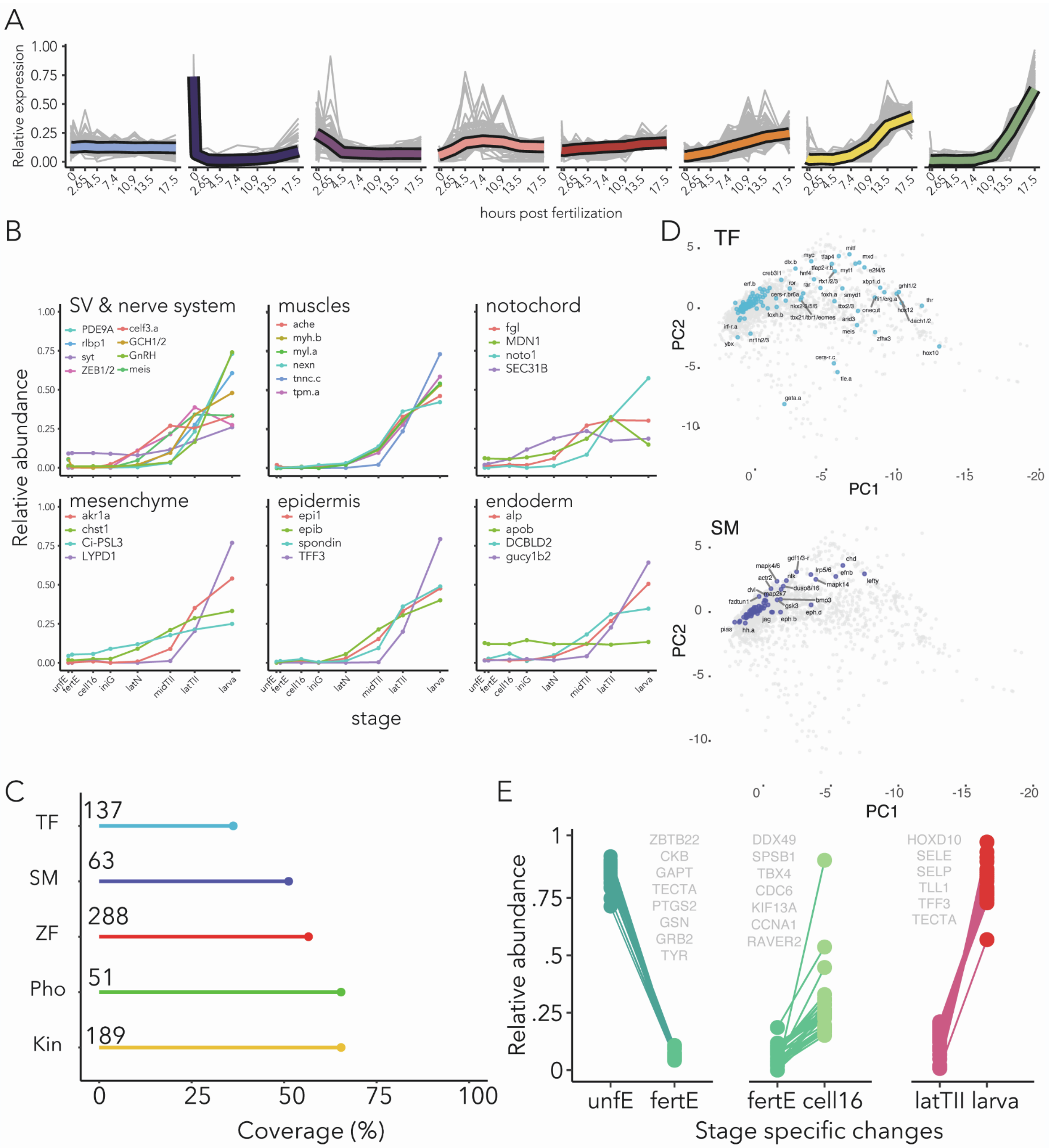

Figure S4 Protein expression dynamics during embryogenesis, related to Figure 2

A, Summary of the temporal pattern of protein expression during embryogenesis. Individual proteins (n = 7,095) are depicted in gray, while the data representing the median for each cluster is superimposed and color-coded.

B, Tissue-level marker protein levels. Metaplots display the temporal dynamics for selected proteins. ‘SV' denotes sensory vesicle. Capital letters represent human orthologs, while lowercase letters indicate *Ciona* gene names.

C, Percentage of all annotated transcription factors (TF), signaling molecules (SM), phosphatases (Pho), and kinases (Kin) detected at the protein level. Numbers near the y-axis denote the number of genes for these protein classes.

D, Principal Component Analysis (PCA) of the expression levels of proteins in the embryo time series. Points on the graph represent individual proteins (7,095), with color coding indicative of different protein classes as in C. A 'salt and pepper' pattern for TFs and SMs is observable.

E, Stage-specific protein differences at fertilization (from unfE to ferE), maternal-zygotic transition (from fertE to cell16), and in preparation for swimming tadpoles (from latTII to larva). Stages are color-coded as in Figure 2A.

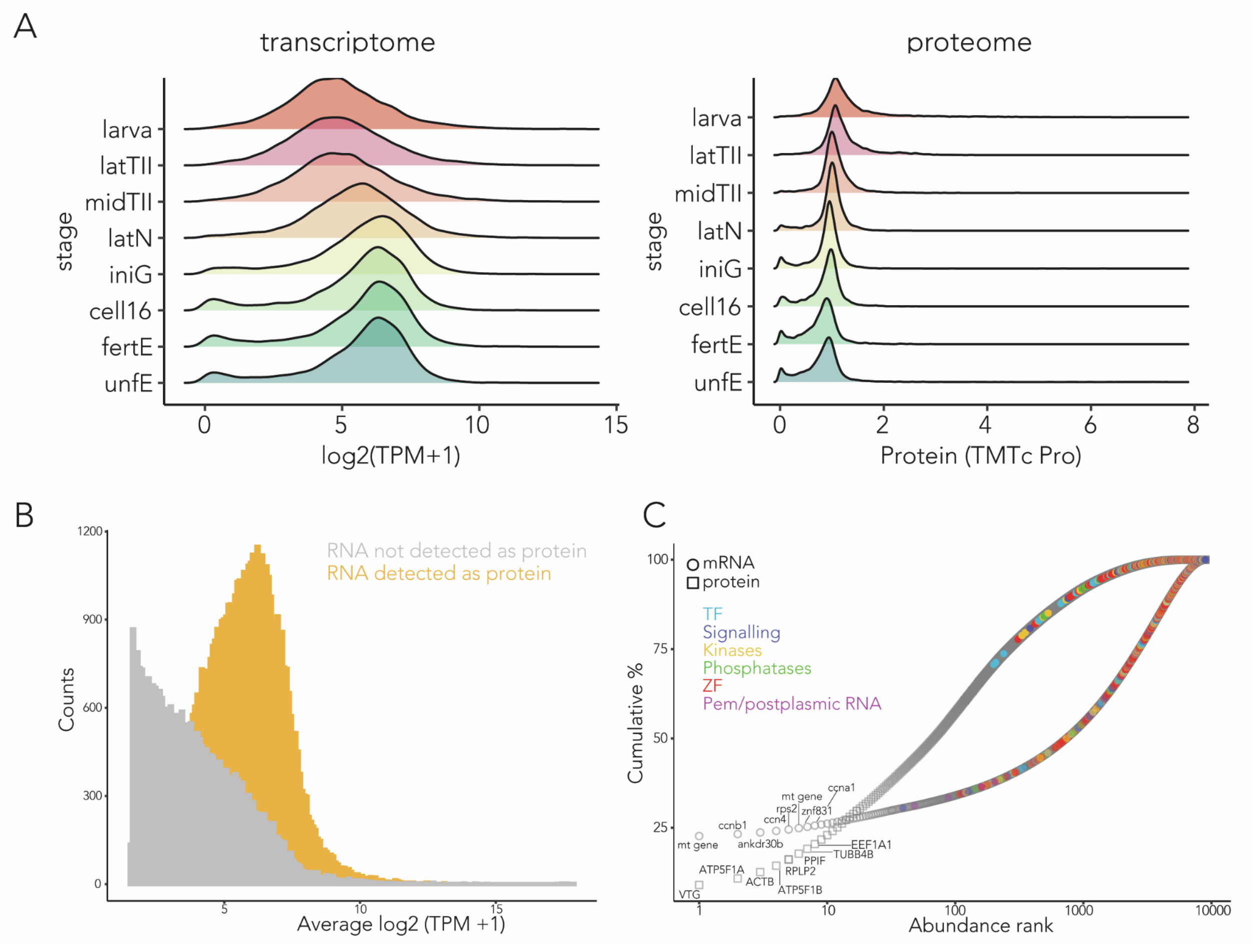

Figure S5 Proteogenomics and dynamic range of transcript and protein expression, related to Figure 2

A, Genes abundance spans a broader range of orders of magnitude compared to protein abundance. In both cases, approximately 90 % of the transcriptome or proteome is concentrated within about three orders of magnitude around the median value.

B, Dynamic range of gene abundance with the proportion of transcripts detected exclusively by RNA-seq (grey) and those also identified at the protein level (yellow). Proteins associated with lower abundance genes are less detected.

C, Cumulative abundance plots of transcriptome (represented by circles) and proteome (represented by squares) ranked by abundance (x-axis), with their respective contributions to the total transcriptome and proteome (y-axis), in the unfertilized egg. The seven most abundant genes and proteins are listed in descending order, these are not the same. Note the protein line rises more quickly than the gene line and the more uniform distribution of transcription factors (TF), signaling molecules, and transcription regulators (kinases, phosphatases, and zinc finger (ZF) genes), within the transcriptome, while these elements are more concentrated at higher ranks in the proteome.

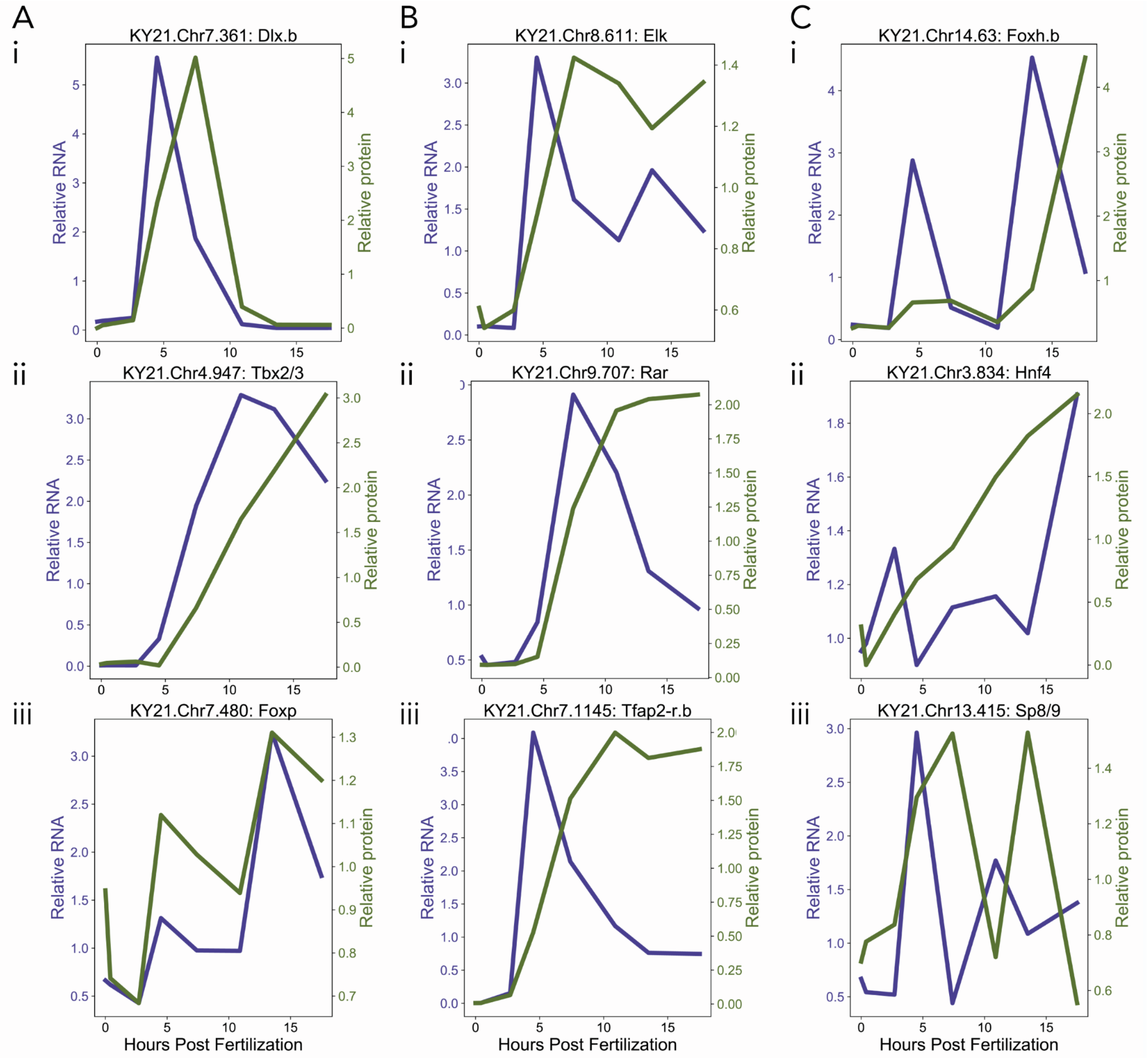

Figure S6 Different RNA and protein dynamics during development for TFs with known involvement in early *Ciona* development, related to Figure 2

A, i-iii Dlx.b, Tbx2/3, and Foxp have similar RNA and protein dynamics, largely matching in relative expression across development.

B, i-iii Elk, Rar, and Tfap2-r.b have similar trends in their respective RNA and protein dynamics, with RNA being expressed earlier than protein and degrading while protein expression remains high.

C, Foxh.b, Hnf4, and Sp8/9 do not have strong trends in their RNA and protein dynamics. RNA and protein expression seem more sporadic, with RNA coming in distinct waves that are not necessarily followed by protein.

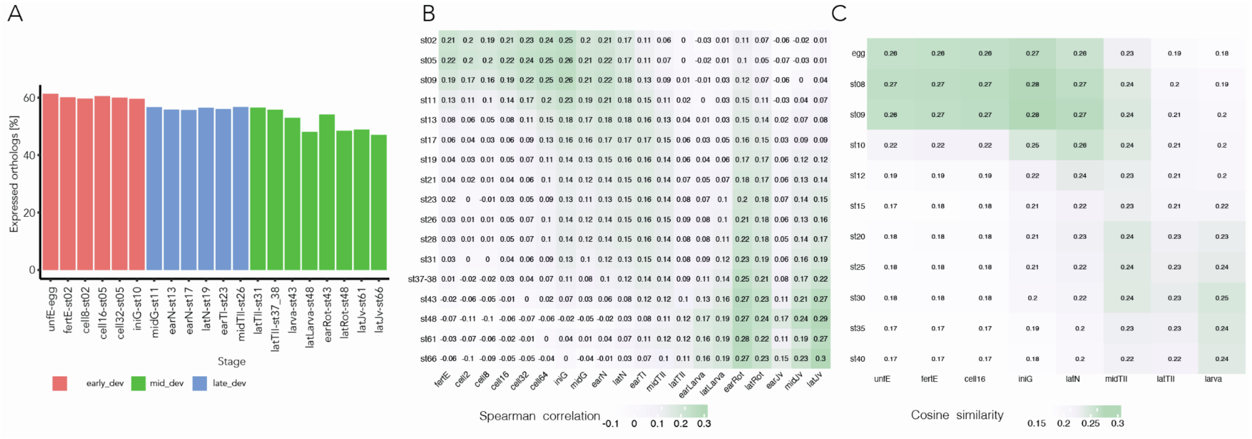

Figure S7 Extended transcriptome analysis, related to Figure 4

A, Comparison of the orthologues temporal associations between *Ciona* and *Xenopus*. These shared genes are more active in the earlier stages.

B, Heatmaps of comparisons of all single copy one-to-one orthologs in an extended transcriptome using Spearman correlation (n=7,636; based on *Xenopus* and *Ciona* data from Hu et al., 2017)

C, Heatmaps of comparisons of all single copy one-to-one orthologs using Cosine similarity (n=7,636; *Xenopus* data from Session et al. 2016 and *Ciona* data from this paper).

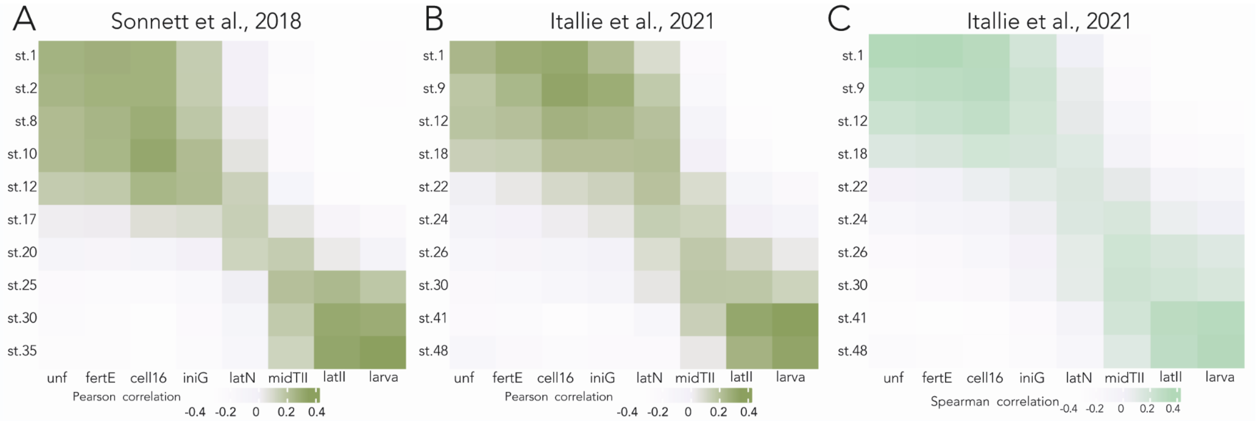

Figure S8 Extended proteome analysis, related to Figure 4

A,B,C, Heatmaps representing Pearson (A, B) and Spearman (C) correlations from pairwise comparisons of one-to-one orthologs data between *Ciona* and *Xenopus*.

Irrespective of the reference frog protein dataset used for comparison against the *Ciona* proteome, a consistent pattern emerges, showing the highest similarity at early and late stages of embryogenesis (compare A to B). The two frog independent time series align at three specific timepoints: st.1, st.12, and st.30. The time series from Sonnet et al., 2018 provides more stages in early development, while that from Itallie et al., 2021, covers more stages in late development, specifically st.41 and st.48.

Regardless of the metric used (Pearson or Spearman) to quantify proteome similarity across species, consistent results are observed (compare B with C) (n protein = 3,350 from Sonnet et al., 2018; n protein = 5,376 from Itallie et al., 2021).

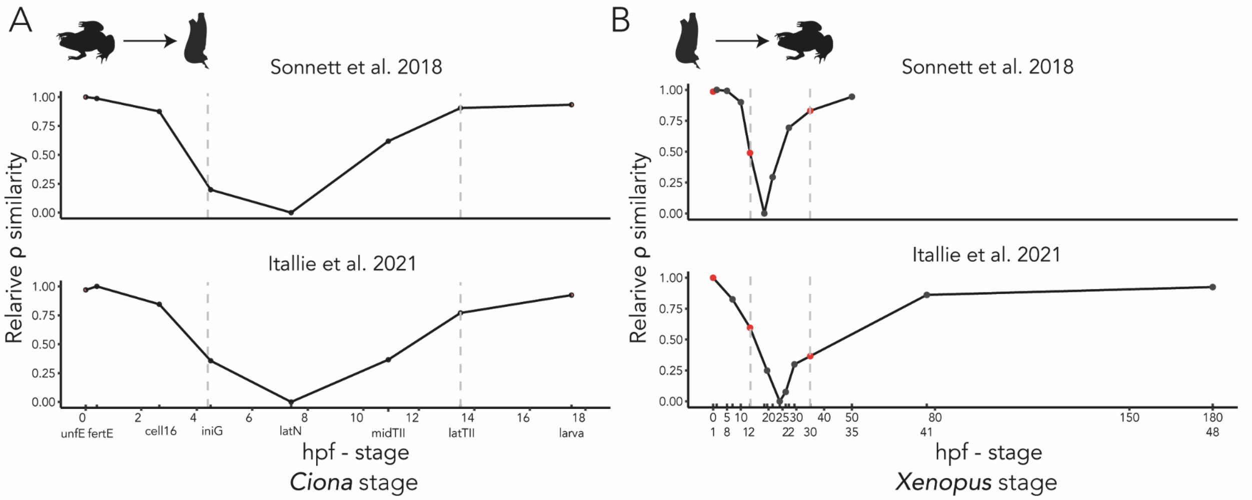

Figure S9 Chordates share minimal proteome similarity during mid-developmental stages, related to Figure 4

A, Spearman's rho proteome similarity, normalized using the minimum and maximum values from all one-to-one ortholog sets, from *Xenopus* to each *Ciona* stage. Regardless of the frog proteome time series (top and bottom), a consistent pattern emerges in this proteome time series, showing minimal similarity at the stage of neurulation (in between vertical grey lines).
B, Normalized Spearman similarity from *Ciona* to two independent *Xenopus* time series (top and bottom), revealing maximal similarity at the onset of embryogenesis and during the tadpole stages. Red points indicate identical frog timepoints in the two time series. Vertical grey lines highlight the developmental window with the highest divergence.

Table S4 *Ciona* RNA-seq alignment statistics, related to Figure 2.

Library	Biological replicate	Mapping rate
unfE	1	94.29%
unfE	2	94.48%
ferE	1	94.49%
ferE	2	94.46%
16-cell	1	94.17%
16-cell	2	94.19%
iniG	1	93.25%
iniG	2	93.22%
Late neurula	1	91.73%
Late neurula	2	91.85%
Mid tailbud II	1	91.00%
Mid tailbud II	2	91.61%
Late tailbud II	1	92.20%
Late tailbud II	2	92.52%
Larva	1	91.64%
Larva	2	91.76%
